# Anti-interference diffractive deep neural networks for multi-object recognition

**DOI:** 10.1038/s41377-026-02188-7

**Published:** 2026-02-03

**Authors:** Zhiqi Huang, Yufei Liu, Nan Zhang, Zian Zhang, Qiming Liao, Cong He, Shendong Liu, Youhai Liu, Hongtao Wang, Xingdu Qiao, Joel K. W. Yang, Yan Zhang, Lingling Huang, Yongtian Wang

**Affiliations:** 1 Beijing Engineering Research Center of Mixed Reality and Advanced Display, School of Optics and Photonics, Beijing Institute of Technology, Beijing, 100081 China; 2 National Key Laboratory on Near-surface Detection, Beijing, 100072 China; 3 Beijing Key Laboratory of Metamaterials and Devices, Key Laboratory of Terahertz Optoelectronics of Ministry of Education, Capital Normal University, Department of Physics, Capital Normal University, Beijing, 100048 China; 4 Qiyuan Lab, Beijing, 100095 China; 5 Engineering Product Development, Singapore University of Technology and Design, Singapore, 487372 Singapore; 6 Department of Electrical and Systems Engineering, University of Pennsylvania, Philadelphia, PA 19104 USA

**Keywords:** Imaging and sensing, Photonic devices

## Abstract

Optical neural networks (ONNs) are emerging as a promising neuromorphic computing paradigm for object recognition, offering unprecedented advantages in light-speed computation, ultra-low power consumption, and inherent parallelism. However, most of ONNs are only capable of performing simple object classification tasks. These tasks are typically constrained to single-object scenarios, which limits their practical applications in multi-object recognition tasks. Here, we propose an anti-interference diffractive deep neural network (AI D^2^NN) that can accurately and robustly recognize targets in multi-object scenarios, including intra-class, inter-class, and dynamic interference. By employing different deep-learning-based training strategies for targets and interference, two transmissive diffractive layers form a physical network that maps the spatial information of targets all-optically into the power spectrum of the output light, while dispersing all interference as background noise. We demonstrate the effectiveness of this framework in classifying unknown handwritten digits under dynamic scenarios involving 40 categories of interference, achieving a simulated blind testing accuracy of 87.4% using terahertz waves. The presented framework can be physically scaled to operate at any electromagnetic wavelength by simply scaling the diffractive features in proportion to the wavelength range of interest. This work can greatly advance the practical application of ONNs in target recognition and pave the way for the development of real-time, high-throughput, low-power all-optical computing systems, which are expected to be applied to autonomous driving perception, precision medical diagnosis, and intelligent security monitoring.

## Abstract (web summary)

Recognizing targets in multi-object scenarios, including intra-class, inter-class, and dynamic interference, but not limited to scenarios with multiple interfering objects or spatially overlapping objects by integrating multi-dimensional optical multiplexing technology.

## Graphical abstract

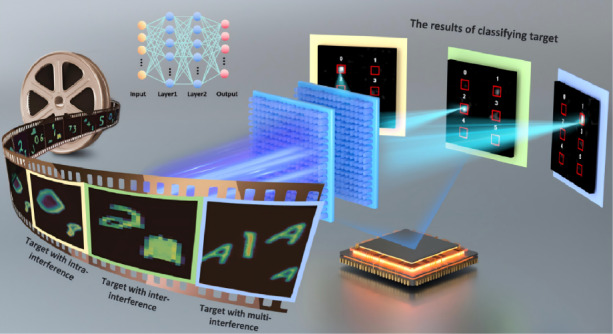

## Introduction

Deep learning techniques have proven to be effective in the classification and localization of objects in multiple scenarios^1^. However, with the increasing complexity of application scenarios, current object recognition technologies face numerous challenges, such as simultaneously detecting multiple objects^2–4^, especially when they occlude or overlap with each other. It is desired to develop a robust model for multi-object detection and instance segmentation capable of handling complex scenarios^5–7^. Besides, certain applications require highly precise recognition of fast-moving objects^8–10^, which requires deep-learning-based high frame-rate processing capability and temporal information modeling ability^11–14^. To meet the above requirements, tensor core processors for object recognition must deliver low latency, high throughput, and exceptional energy efficiency^15^. Traditional digital computers, however, face limitations in speed and energy due to Joule heating, electromagnetic crosstalk, and parasitic capacitance^15–18^. Photonic technologies offer unparalleled advantages in the development of artificial intelligence hardware, providing solutions to overcome the bottlenecks of electronic systems^19^.

Optical neural networks (ONNs) represent a promising neuromorphic computing paradigm for object recognition, enabled by their unique advantages in light-speed computation, ultra-low power consumption, and inherent parallelism. As a key milestone in the advancement of photonic technology, ONNs are drawing increasing attention^20–27^. In recent years, fundamental breakthroughs across diverse domains have accelerated the development of ONNs. Firstly, multi-dimensional multiplexing and interweaving techniques^28^, including wavelength^28–33^, polarization^34–36^, and orbital angular momentum^37,38^, enable massively parallel information processing^39^. Meanwhile, energy-efficient materials and components, such as non-volatile phase-change material in a micro-ring resonator array^40–43^, low-loss lithium niobate modulators^44,45^ and hybrid optoelectronic chips eliminating optical-electrical conversion^46^, pave the way for the development of ONNs with significantly reduced system-level power consumption. Moreover, partially coherent optical neural networks can be implemented by reducing the coherence in either the temporal^47^ or spatial domain^48^, which has been shown to enhance experimental accuracy and robustness. Most notably, novel ONN architectures and training strategies are emerging^49–52^, such as integrated diffractive-interference hybrid design^53^, distributed computing architecture^53,54^, and fully forward mode training methods^55^, further enhancing inference performance and expanding functional diversity and complexity.

However, current ONNs are primarily designed for classifying a single target^56,57^, which is far from the multi-object scenarios commonly encountered in real-world applications. Although some studies have investigated multiplexing schemes to facilitate multi-object classification^30^, these schemes impose strict constraints on the location, size, and category of objects, thereby limiting the flexibility and practicality of the network. To overcome these limitations, several hybrid optoelectronic architectures have been proposed for dynamic scenes^58,59^. However, these architectures require an electronic neural network as a post-processing module to further process the low-dimensional features extracted by ONNs. The need for analog-to-digital conversion introduces latency and increases power consumption, thus compromising the intrinsic advantages of optical computing.

**Fig. 1:**
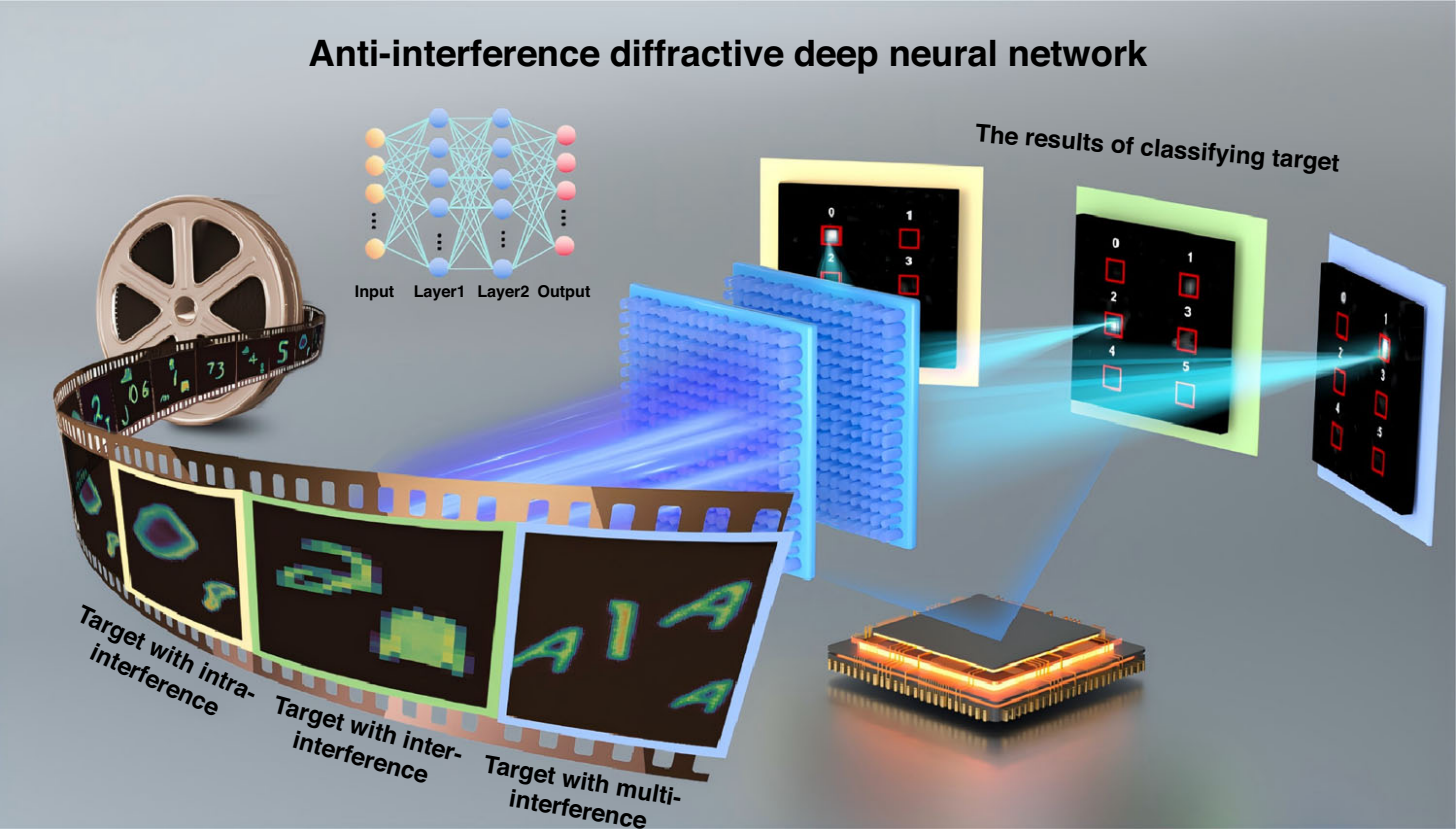
Schematic illustration of Anti-Interference Diffractive Deep Neural Network (AI D^2^NN). The network can classify handwritten digits (0–5) in multi-object scenarios, including intra-class interference, inter-class interference, and dynamic interference

Here, an anti-interference diffractive deep neural network (AI ${{\rm{D}}}^{2}{\rm{NN}}$) consisting of two transmissive diffractive layers is proposed, as schematically illustrated in Fig. 1. We employ distinct deep-learning-based training strategies to distinguish target with interference, and eliminate the impact of undesired interference on target’s recognition result. In this work, targets are defined as handwritten digits 0–5 from Modified national institute of standards and technology (MNIST) dataset. To enhance the network’s robustness against diverse forms of interference, an extensive interference dataset is utilized for training. This dataset includes intra-class interference from other handwritten digits 6–9 from MNIST dataset, inter-class interference derived from the Fashion-MNIST and EMNIST datasets. We also introduce dynamic interference by combining all of the aforementioned categories (40 categories in total) without any constraints on object location or size. The trained network can apply to multi-object scenarios, accurately and robustly recognizing targets and achieving a simulated blind testing accuracy of 87.4%. Furthermore, silicon-based metasurfaces are fabricated to physically implement the proposed AI D^2^NN. We establish a terahertz (THz) experimental platform with 0.85 THz as the incident light source. Using this setup, the AI D^2^NN achieves a blind testing accuracy of 86.7%, which is in good agreement with the numerical simulations.

The presented framework exhibits excellent scalability and can be scaled physically to operate across a broad wavelength range of electromagnetic waves, simply by scaling the diffractive features in proportion to the wavelength range of interest. In addition, it can be seamlessly integrated with optical multi-dimensional multiplexing technologies, enabling more flexible and higher-capacity parallel recognition tasks for multiple targets. Therefore, it can greatly advance the practical application of ONNs in target recognition and pave the way for the development of real-time, high-throughput, low-power all-optical computing systems, which are expected to be applied to autonomous driving perception, precision medical diagnosis, and intelligent security monitoring.

## Results

### Design of AI D^2^NN

**Fig. 2:**
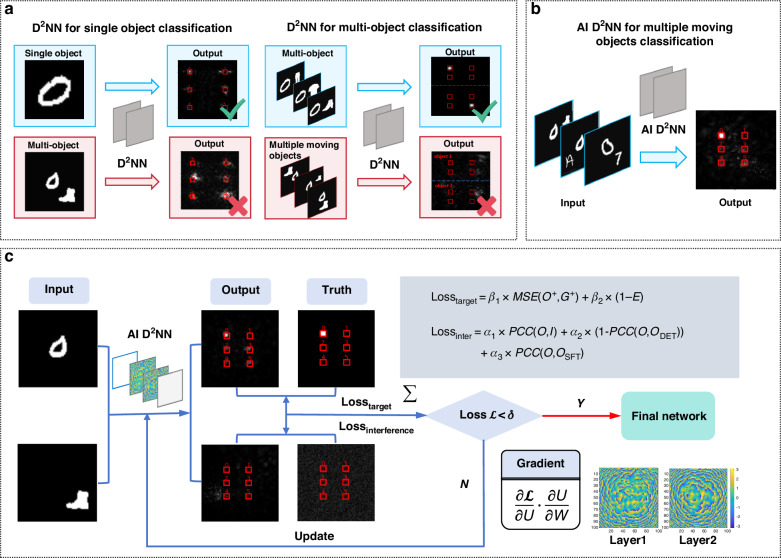
The principle of AI D^2^NN. **a** Limitations of traditional D^2^NN when solving multi-object classification tasks and dynamic object classification tasks**. b** The proposed AI D^2^NN is capable of performing six-class classification of handwritten digits (0–5) in the presence of multiple moving undesired objects**. c** Training schematic of AI D^2^NN

Current ONNs demonstrate reliable performance for single-object recognition or spatially constrained multi-object detection tasks, but exhibit substantial performance deterioration when applied to scenarios exceeding their functional scope, as shown in Fig. 2a. The proposed AI D^2^NN is capable of performing six-class classification of handwritten digits (0–5), even in the presence of undesired objects, as illustrated in Fig. 2b. To achieve this, the basic training schematic is shown in Fig. 2c. A customized training dataset was built by combining handwritten digits 0–5 with an interference set consisting of handwritten digits 6–9, fashions, and letters. Incorporating variability into the training dataset is intended to enhance the network’s robustness in recognizing target objects, enabling selective identification even in complex scenarios with interference across categories, sizes, and positions.

Next, an all-optical neural network was constructed, which is composed of an input layer, multiple diffractive layers and an output layer. The diffractive layers function similarly to hidden layers in fully connected electronic neural networks, processing input information in a linear manner. Each unit within these layers performs complex amplitude modulation of the optical field, serving as a neuron node. Inspired by the Huygens-Fresnel diffraction principle, each neuron receives information in the form of a superposition of secondary optical fields generated by all neurons in the preceding layer, thereby tightly linking the layers and facilitating the hierarchical computation within the optical neural network. More detailed principal illustration of D^2^NN can be found in Supplementary Note 1.

During training, the data was first fed into the network, and the output was obtained via optical diffraction calculations. The loss value was computed based on the loss function, as guidance through back-propagation for fine-tuning the weights of each hidden layer. This procedure highlights the importance of loss function design, and for this task, the loss function was specifically tailored according to different labels.

For label 0–5, a conventional Mean Squared Error (MSE) loss function was employed to maximize the light intensity in the classification region aligned with the target category and minimize the intensity in other classification regions. Moreover, constraint on the light field intensity was imposed to improve the energy efficiency E within the correct classification region.

1 $$Los{s}_{target}={\beta }_{1}\times MSE({O}^{+},{G}^{+})+{\beta }_{2}\times (1-E)$$

For label 6, the output optical field of interference is uniformly distributed as random noise outside the classification regions, which was achieved by designing a loss function based on the Pearson Correlation Coefficient *(PCC)*. *PCC* quantifies the degree of linear dependence between two variables, with values ranging from -1 to 1. An absolute value approaching 1 indicates a strong linear correlation, while a value of 0 signifies no linear relationship, which can be expressed mathematically as:

2 $$PCC(X,Y)=\frac{{\mathrm{cov}}(X,Y)}{{\sigma }_{X}{\sigma }_{Y}}=\frac{{\sum }_{i=1}^{n}({X}_{i}-\bar{X})({Y}_{i}-\bar{Y})}{\sqrt{{\sum }_{i=1}^{n}{({X}_{i}-\bar{X})}^{2}}\sqrt{{\sum }_{i=1}^{n}{({Y}_{i}-\bar{Y})}^{2}}}$$

The loss function for label 6 was designed as a weighted combination of three PCC metrics:

3 $$Los{s}_{inter}={a}_{1}\times PCC(O,I)+{a}_{2}\times (1-PCC(O,{O}_{DET}))+{a}_{3}\times PCC(O,{O}_{SFT})$$

Here, *O* and *I* mean optical field distribution in output plane and input plane separately. *O*_*DET*_ means optical field avoiding all classification regions. *O*_*SFT*_ means optical field which shifts several pixels. *PCC (O, I)* aims to minimize the similarity between the input and output, preventing the direct transmission of interference information to the output. *PCC (O, O*_*DET*_*)* guides the spatial distribution of the output optical field to bypass the detection region when this value trends toward 1, and the *PCC (O, O*_*SFT*_*)* is minimized to force the D^2^NN to generate uninterpretable noise-like output patterns.

Within each batch, the total loss was computed by first calculating the loss for each label separately and then summing these label-specific losses.

4 $$Loss=Los{s}_{target}+Los{s}_{inter}$$

The phase parameters of diffractive neurons were optimized using the backpropagation algorithm and stochastic gradient descent. After several epochs of training, the model converged and generated desired optical field distribution. More detailed information about training procedures and energy calculation methods are shown in Supplementary Note 2 and Supplementary Note 6.

To assess the classification accuracy of the network, a selective evaluation approach was designed. For digits 0–5, the classification was considered correct if the detection region with the maximum intensity corresponds to the correct label. For interference labeled 6, the recognition was deemed correct if both the mean and standard deviation of the optical output field were less than 0.2. This way provided an intuitive measure of the network’s capability to suppress the influence of light field interference on digit classification results.

**Fig. 3:**
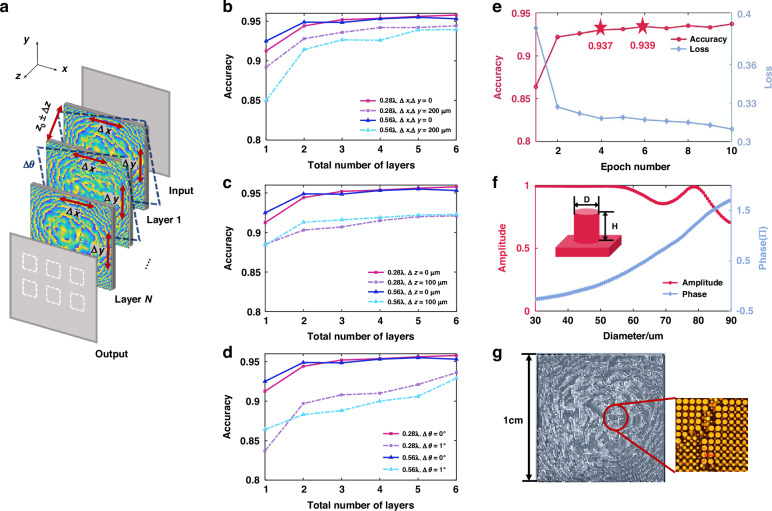
Training results of numerical network and optimization of microstructure parameters. **a** Different error sources in the experiment. **b**–**d** Comparison of training accuracy for networks with neuron sizes of 100 µm and 200 µm under interlayer transverse shift errors, z-axis displacement errors and rotational misalignments as the network depth increases. **e** Training accuracy and loss of dual-layer AI D^2^NN under 1–10 training epochs. **f** Complex amplitude response of microstructure of different diameters under 0.85 THz. **g** General layout of the fabricated metasurface and zoom-in microscopic images of meta-atoms

Additionally, other strategies were implemented to enhance the network’s adaptability to experimental conditions. A 0.85 THz (352 µm) laser light source was employed for subsequent experiments. The impact of diffractive layer number, neuron sizes, and training iterations on network performance were systematically investigated. The diffractive surfaces, each comprising a 100 × 100 neuron array with respective neuron sizes of 100 µm and 200 µm, were compared under various experimental error conditions, including transverse shifts (within 200 µm), rotational misalignments (within 1 radian), and displacement errors along the z-axis (within 100 µm), as shown in Fig. 3a. When trained in the absence of random errors, both networks exhibit comparable classification accuracies. However, the performance of these two networks degrades to different extents when introducing aforementioned experimental errors during training, as illustrated in Fig. 3b–d. It can be concluded that the network with neuron sizes of 100 µm exhibits greater robustness compared to 200 µm counterpart. Considering fabrication constraints and alignment difficulties, a neuron size of 100 µm and a dual-layer configuration were selected for subsequent experiments. Subsequently, under the above network configuration, the effect of training epochs (from 1 to 10) on classification accuracy was further assessed, as shown in Fig. 3e and Table S1 in Supplementary Note 2. After only 4 training epochs, the network achieves an accuracy exceeding 93.7%, which remains within the range of 93.7% to 94% in the subsequent epochs. Details about the combined effects of multiple experimental errors on network performance are available in Table S3 in Supplementary Note 3.

### Design of the metasurfaces and elementary microstructures

As a proof of concept, the metasurface structures were designed for neuron phase modulation. The metasurface consists of high-impedance silicon cylinders deliberately fabricated on a silicon substrate. The electromagnetic response of each microstructure was calculated using the finite-difference time-domain (FDTD) method. The height of the microstructure was fixed at 240 µm, with a periodicity of 100 µm in both x and y directions. The incident wavelength was set at 0.85 THz.

To explore the phase modulation properties, the diameters of the microstructures were varied from 30 µm to 90 µm in 300 nm increments. This yielded the complex response coefficients of the microstructures under x-polarized incident light, as shown in Fig. 3f. The 16 types of structures with amplitude coefficients close to 1 and phase responses nearest to integer multiples of π/8 were selected. The specific structural parameters are listed in Table S4 in Supplementary Note 5. These structures were then assembled according to the neuron values of the trained diffractive neural network. Ultimately, two metasurfaces of 100 × 100 pixels were designed and fabricated for subsequent terahertz experiments. The overall distribution of the fabricated metasurface and the zoom-in optical microscopic images of metasurface are shown in Fig. 3g.

### Anti-interference classification results

The proposed network is capable of accurately classifying the target in multi-object scenarios. Specifically, when the input contains both interference and target objects, the AI D^2^NN identifies the target, as evidenced by the concentration of energy within a designated region on the output plane. To evaluate the network’s performance, both numerical simulations and experimental evaluations were conducted.

**Fig. 4:**
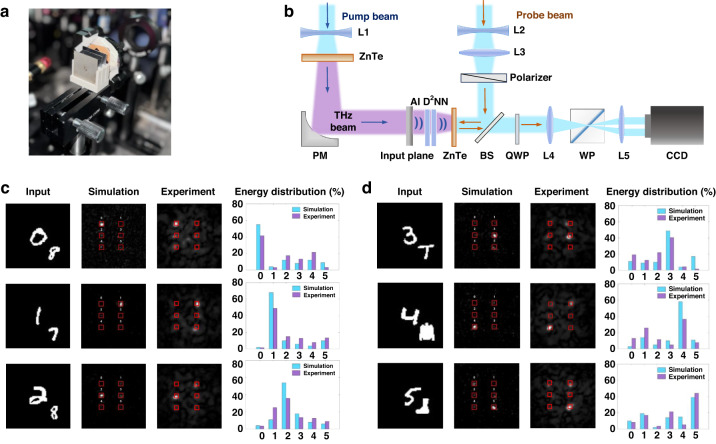
Anti-Interference Classification Results. **a**, **b** Experimental ONN set up. **c** The results of AI D^2^NN classifying digits and intra-class interference. **d** The results of AI D^2^NN classifying digits and inter-class interference

In the numerical simulations, a test dataset of 6000 images containing digits and interference was built. These images were input into the trained network model to assess the classification accuracy of the primary digits. In the experiment, the input mask and two diffractive layers were assembled using 3D-printed holder, as shown in Fig. 4a, completing the physical cascading of the AI D^2^NN. A terahertz experimental platform was established, depicted in Fig. 4b, and 60 samples were tested to evaluate the accuracy of the physical network. Detailed experimental set-up and fabricated samples are presented in Fig. S1 in Supplementary Note 4.

First, the network’s classification capability in the presence of target digits 0–5 and intra-class interference digits 6–9 was evaluated. This case can mimic real-world industrial scenarios, such as automated inspection of specific types of components on a production line or the identification of vehicle types in transportation systems. The corresponding confusion matrix of the test dataset is shown in Fig. S3a in Supplementary Note 7, which indicates that the network achieved a recognition accuracy of 90.1% for digits 0–5 under scenes with intra-class interference. Figure 4c demonstrates numerical simulation results and experimental results, which exhibit high consistency, proving that the delicately designed ONN metasurfaces possess the capability for target recognition.

Next, the network’s classification capability was assessed when target and inter-class interference appear together. According to the confusion matrix in Fig. S3b in Supplementary Note 7, the network’s recognition accuracy for digits 0–5 reaches 89.7% in the presence of inter-class interference. Both numerical simulation and experimental results are shown in Fig. 4d. The energy distribution demonstrates that the system effectively concentrates most of the energy at the correct classification region. Although light spots appear in other classification regions, the maximum optical field intensity in these regions is significantly lower than that in the correct region, with a ratio ranging from approximately 0.48 to 0.70. Furthermore, no significant light spots appear outside the classification regions, indicating that the network can accurately classify target while remaining resistant to the influence of other interference present in the scene.

**Fig. 5:**
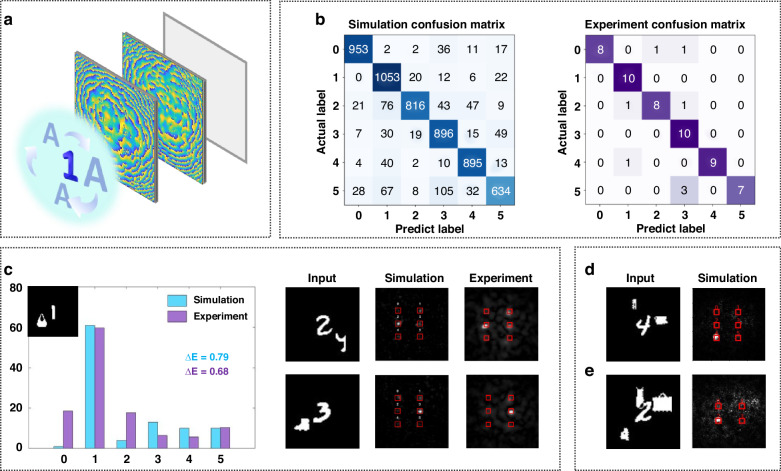
Anti-interference classification capability under dynamic multi-object scenes. **a** AI D^2^NN under dynamic testing environment. **b** Confusion matrices for the numerical and experimental results. **c** Energy distribution of numerical and experimental results. **d** Classification results when scenes containing one target and two moving interferences. **e** Classification results when scenarios with one target and more than two moving interferences

To better simulate real-world scenarios, the dynamic multi-object scenes were constructed, where the target remains stationary while interfering objects allocate randomly in three-dimensional space. This configuration imposes natural challenges including objects clustering, partial occlusions, and scale variations, all arising organically from the objects’ movement. During the dataset construction process, we simulated the movement of interfering objects parallel to the detection plane by varying their positions in the image, and simulated their movement perpendicular to the detection plane by scaling the interfering objects. As shown in Fig. 5a, by randomly altering the sizes and positions of the interfering objects, a series of dynamic multi-object scenarios were generated. The test results for representative examples of correct and incorrect identifications are presented in Fig. 5b. In numerical simulations, the network achieved 87.4% accuracy on a test set of 6000 images. In experimental validation, it attained 86.7% accuracy with 10 samples per class, closely aligning with the simulation results and demonstrating the robustness of the design.

In addition, metrics such as signal-to-noise ratio *(SNR)* and the discrimination factor (*ΔE*) were incorporated into the network evaluation metrics. In this study, *SNR* is defined as the ratio of the maximum average intensity of the classification regions to the average intensity of the output plane outside the six detection regions, while *ΔE* represents the ratio of the difference between the highest and second-highest energies across the six detection regions to the highest energy. As presented in Fig. 5c, numerical simulations yielded an *SNR* of approximately 31 and a *ΔE* value close to 0.8, while experimental measurements showed an SNR of around 23 and a *ΔE* of 0.68. Furthermore, Fig. 5d shows the network’s output for the flexible test cases, demonstrating that the network can accurately classify the primary target regardless of the location and size of the interference. Besides, video streams were leveraged, which contains target (digit 4) and persistently moving interference as input. The corresponding output performance confirms the network’s classification capability under dynamic scenes. The input and output videos are presented in Supplementary Video 1 and Fig. S5 in Supplementary Note 7.

The concept of transforming the optical fields of interfering objects into background noise through ONN is inherently extendable. Even as the number and categories of interference increase, the network still demonstrates excellent capability in classifying the primary target in multi-object scenarios. In theory, more complex scenarios for target recognition can be achieved by employing more diverse and extensive datasets, more rigorous loss functions and deeper network models during training phase. Therefore, we successfully constructed scenarios with one target and more than two interfering objects, in which the positions and sizes of all interfering objects vary flexibly. The model’s target recognition accuracy and the output optical field distribution were evaluated using numerical simulations, as shown in Fig. 5e, Figs. S6–S12 in Supplementary Note 7 and Figs. S15–S17 in Supplementary Note 8, which demonstrated AI D^2^NN has strong adaptability to complex scenarios. Lastly, a 50-frame video of the input and corresponding output is shown in Supplementary Video 2. The input scene contains a target (digit 2) and three interfering objects moving along randomly generated trajectories with varying sizes, further showcasing the network’s scalability and generalization capability.

## Discussion

Most existing ONNs are designed for single-object classification, and their performance deteriorates significantly when multiple objects present in a scene. To overcome this limitation, we proposed an anti-interference diffractive deep neural network for multi-object environments. Our approach enables the network to distinguish between target and interferences, and focus target’s intensity onto designated detection region while transforming interferences’ intensity into low-energy and uniformly distributed noise. This training strategy demonstrated strong robustness, achieving a numerical accuracy of 87.4% even in scenarios involving 6 types of targets and 40 types of dynamic interference. Furthermore, we constructed a terahertz-based diffractive neural network for experimental validation. The measured classification accuracy closely matched the simulation results, highlighting the network’s potential for rapid and intelligent identification of primary targets in complex environments.

Future work can be developed regarding functional capability expansion, operational wavelength scaling and system-level integration. By integrating multi-dimensional optical encoding technology with AI D^2^NN, each channel can be dedicated to recognizing only specific object classes while resisting interference from other objects, thus enabling multi-object recognition, as shown in Fig. S19. In addition, by incorporating spatially invariant systems such as optical convolution operators, the AI D^2^NN can be extended to handle dynamic target recognition based on the convolution results. Besides, the operating wavelength of the network can be expanded to visible or near-infrared spectral range for broader applications. Further optimization such as energy efficiency, optical loss, and fabrication precision will be required for practical implementation, as discussed in Table. S6 in Supplementary Note 9. Furthermore, by capitalizing on the lightweight and planar advantages of metasurface, we can integrate metasurface with the mature complementary metal-oxide semiconductor imaging sensor, to fabricate an integrated and compact AI D^2^NN system. Overall, AI D^2^NN will accelerate their transition from laboratory prototypes to real-world applications, demonstrating strong potential for deployment in autonomous driving perception, precise medical diagnostics, and intelligent security monitoring.

## Materials and methods

### Numerical calculations

We employed the finite-difference time-domain (FDTD) method to simulate the optical response of microstructures with varying size. The modeled structures were subwavelength cylindrical rods made of high-impedance silicon (refractive index of 3.45), illuminated by a linearly x-polarized plane wave at 0.85 THz. The Si substrate thickness was set to 760 µm, and the height of each rod was fixed at 240 µm. The diameter of the microstructures was varied from 30 µm to 90 µm in 300 nm increments. Periodic boundary conditions were applied in the x and y directions with a unit cell period of 100 µm to model an infinite array. Along the z-direction, a perfectly matched layer (PML) was employed to absorb outgoing waves and eliminate artificial reflections. To characterize the optical response, a point monitor was placed to record the transmitted wave phase, while a planar field positioned above the silicon rods captured the transmission intensity. By analyzing both phase and transmittance, a comprehensive insight into how rod’s size interact with the incident terahertz waves were gained.

### Training of the AI D^2^NN

The numerical model of the proposed AI D^2^NN was deployed on a computer for training. The hardware configuration includes an NVIDIA GeForce RTX 4060 GPU and an Intel® Xeon® Gold 6248 R CPU. The software environment utilized Python 3.8.17 and PyTorch 2.0.1+cu117.

A dataset consisting of 40,000 handwritten digit images and interference images was used for training, with a batch size of 8 and a learning rate of 0.01. During training, the Mean Squared Error (*MSE*) loss function and the Pearson Correlation Coefficient (*PCC*) was selected to calculate loss values, these metrics are commonly used for target classification and imaging quality assessment in machine learning task. The Adam optimizer was employed to fine-tune the phase parameters of each layer based on the computed gradients. Above process is repeated many times until the network converged.

### Experimental setup

The AI D^2^NN was experimentally implemented within a terahertz time-domain spectroscopy (THz-TDS) system. Femtosecond laser pulses were split into pump and probe paths: the pump beam generates terahertz radiation, while the probe beam was used to record the transmitted terahertz waveform after passing through the network. A CCD electro-optic sampling system (MER-531-20GM-P) was employed to scan the terahertz pulses, enabling the Fourier transformation of the recorded time-domain signals to obtain their spectral components in the frequency domain. To produce an ideal input intensity profile, a stainless-steel mask was used to block portions of the terahertz beam, creating a customized spatial intensity distribution. The custom 3D-printed holder (printer model: JG-A240, fabrication accuracy: 100 µm) ensured precise alignment and interlayer spacing between the mask and diffractive layers.

## Supplementary information

Supplementary Information for Anti-Interference Diffractive Deep Neural Networks for Multi-Object Recognition

The network’s classification capability under dynamic scenes

The network’s classification capability under complex scenes

## Data Availability

All data needed to evaluate the conclusions in the paper are present in the paper and/or the Supplementary Materials.
