# Supplementary material for: Anti-interference diffractive deep neural networks for multi-object recognition
Source: Light Sci Appl. 2026 Feb 3;15:101. doi: 10.1038/s41377-026-02188-7 (PMC12868834; doi:10.1038/s41377-026-02188-7)
Supplement: Supplementary file 1 — Supplementary Information for Anti-Interference Diffractive Deep Neural Networks for Multi-Object Recognition [file 41377_2026_2188_MOESM1_ESM.docx]

Supplementary Information for

Anti-Interference Diffractive Deep Neural Networks

for Multi-Object Recognition

Zhiqi Huang^1,2,#^, Yufei Liu^3,#^, Nan Zhang^1,2,#,*^, Zian Zhang^1,2^, Qiming Liao^1,2^, Cong He^1,2^, Shendong Liu^1,2^, Youhai Liu^4^, Hongtao Wang^5^, Xingdu Qiao^6^, Joel K. W. Yang^5^, Yan Zhang^3,*^, Lingling Huang^1,2,*^, Yongtian Wang^1,2^

^1^Beijing Engineering Research Center of Mixed Reality and Advanced Display, School of Optics and Photonics, Beijing Institute of Technology, Beijing, China.

^2^National Key Laboratory on Near-surface Detection, Beijing, China.

^3^Beijing Key Laboratory of Metamaterials and Devices, Key Laboratory of Terahertz Optoelectronics of Ministry of Education, Capital Normal University, Department of Physics, Capital Normal University, Beijing, China.

^4^Qiyuan Lab, Building 8, No. 55 Zique Road, Haidian District, Beijing, China.

^5^Engineering Product Development, Singapore University of Technology and Design, Singapore, Singapore.

^6^Department of Electrical and Systems Engineering, University of Pennsylvania, Philadelphia, PA 19104, USA.

^#^ These authors contributed equally: Zhiqi Huang, Yufei Liu, Nan Zhang

^*^ E-mail: nanzhang@bit.edu.cn; yzhang@cnu.edu.cn; huanglingling@bit.edu.cn

This file includes:

Supplementary Note 1: Angular spectrum diffraction method

Supplementary Note 2: The training of AI D^2^NN

Supplementary Note 3: Analysis of robust training

Supplementary Note 4: Detailed experimental setup and fabricated samples

Supplementary Note 5: The structural parameters of cylindrical rods

Supplementary Note 6: Energy distributions for the AI D^2^NN

Supplementary Note 7: Trained network’s classification results of different cases

Supplementary Note 8: Analysis of the Effect of Different Interference on Network’s

Classification Performance

Supplementary Note 9: The improvement for network’s classification performance

**Supplementary Note 1: Angular spectrum diffraction method**

In AI D^2^NN, each meta-unit behaves like a separate neuron in a neural network, interconnecting with the meta-units of the previous and next layers by diffraction of light. This physical process can be described by a mathematical model through the angular spectrum method^1^.

Assume the complex amplitude distribution of input plane is *U* (*x, y,0*), field distribution at distance z is *U (x, y, z),* which can be expressed using angular spectrum diffraction method.

$U\left(x,y,z\right)=F^{-1}\left\{F\left[U\left(x,y,0\right)\right]\cdot H\left(f_{x} ,f_{y },z\right)\right\}$ (S1)

Here, *F* and *F⁻¹* denote the Fourier transform and inverse Fourier transform, respectively, and *H* represents the transfer function. The angular spectrum method decomposes the complex amplitude distribution on the input plane into plane waves of different spatial frequencies through the Fourier transform. Each component’s optical field after propagation along the axial distance *z* is calculated separately, and the spatial distribution of the optical field at distance *z* is obtained through the inverse Fourier transform. In this process, the transfer function can be expressed as:

$H\left(f_{x} ,f_{y },z\right)=e^{ik_{z} z}$ (S2)

Here, *k_z_* is the wave vector component along the propagation direction. This transfer function describes the change of complex amplitude as a plane wave propagates along the *z*-axis. Meanwhile, the optical field is further modulated by the diffractive layer neurons during propagation, which is represented as the product of the complex amplitude transmission coefficient and the optical field, the mathematical expression is as follows:

$\text{u}_{\text{n}}^{l+1}(x,y)=\iint_{s} F^{-1}\{F\{u_{n}^{l}(x_{n,}y_{n})t_{n}^{l}(x_{n},y_{n})\}H(f_{x},f_{y},z)\}$ (S3)

Therefore, the input optical field at node n of the next layer *l+1* is obtained by receiving and summing up all the secondary optical fields generated by the neurons in the previous layer *l*. Repeating the process described in formula S3, a multi-layer diffractive neural network is implemented, making the multi-layer architecture distinct from a single-layer network^2^. It should be noted that $t_{n}^{l}(x_{n},y_{n})$represents the complex - amplitude modulation of the optical field by the meta-unit, since we select micro-pillar structures with a transmittance close to 1, $t_{n}^{l}(x_{n},y_{n})$can be simplified to , and subsequent optimizations are also performed specifically for .

**Supplementary Note 2: The training of AI D^2^NN**

This section provides further details to supplement the discussion of network principles presented in the main text. To enable optical neural networks (ONNs) to achieve target classification in multi-object scenarios, we focus on specific designs in datasets construction and loss function configuration.

1. Dataset

Both the training dataset and validation dataset consist of two parts: a target dataset, which is a subset (digits 0-5) of the MNIST training set containing 36,000 samples, and an interference dataset, which include sample from the MNIST digits 6-9, the Fashion-MNIST dataset (10 categories) and the EMNIST dataset (26 categories), with a total of 128,000 samples.

Distinct preprocessing pipelines are employed for the target and interference datasets.

For target dataset: Each image (28×28 pixels) is binarized and centered within an all-zero background of 56×56 pixels, and assigned its corresponding label (0–5).

For interference dataset: Each image (28×28 pixels) is binarized and randomly scaled from 10×10 pixels to 28×28 pixels. The resized image is then placed at a randomly selected position within an all-zero 56×56 background, and assigned the label as 6.

After that, both the target dataset and the interference dataset are split at a ratio of 5:1 to form the training and validation datasets.

Finally, 20000 target samples and 20000 interference samples are randomly selected from the training dataset and shuffled to form the final training dataset, while 5,000 target samples and 5,000 interference samples from the validation datasets are used for model validation.

1. Loss function

We designed separate loss functions for the target and the interference. For the target loss function, the index of the classification region with the highest intensity on the output plane is required to match the label. To achieve that, the difference between the optical field intensity among the six classification regions and the one-hot encoding of the target label is computed using Mean Squared Error (*MSE*) loss function. Meanwhile, to ensure the energy is concentrated as much as possible in the classification regions, we calculated the ratio of the maximum energy among the six classification regions to the total energy sum of the six regions, and make it close to 1 during the optimization process. The mathematical expression is as follows:

$Loss_{target}=\beta_{1}\times MSE(O^{+},G^{+})+\beta_{2}\times(1-E\text{）}$ (S4)

For the interference loss function, we employed Pearson Correlation Coefficient (*PCC*) to make interference output field distribution like random noise outside the classification regions^3^. The components of this loss function are discussed specifically in the main text. The mathematical expression is as follows:

$Loss_{inter}=a_{1}\times PCC(O,I)+a_{2}\times\text{（}1-PCC(O,O_{DET})\text{）}+a_{3}\times PCC(O,O_{SFT})$ (S5)

Here, *O* and *I* mean optical field distribution in output plane and input plane separately, *O_DET_* means optical field avoiding all classification regions, *O_SFT_* means optical field shifts several pixels.

Each training batch consists of 8 images, which include both targets labeled as 0 to 5 and interference labeled as 6. Different calculation methods for the loss function were selected according to the labels, and all the loss values in one batch were summed up.

$Loss=Loss_{target}+Loss_{inter}$ (S6)

The stochastic gradient descent (SGD) algorithm and the Adam optimizer was employed to update the phase value of each layer in the network. During the optimization process, the loss gradually decreases, and ultimately the expected output optical field effect is achieved.

Each epoch for our proposed D^2^NN takes approximately 12 minutes. The detailed loss and accuracy values over 10 epochs is provided in Table S1:

**Table S1. Training results of AI D^2^NN over 10 epochs**

| Epoch number | Training Accuracy % | Loss |
| --- | --- | --- |
| 1 | 86.4 | 0.388 |
| 2 | 92.2 | 0.327 |
| 3 | 93.0 | 0.322 |
| 4 | 93.7 | 0.318 |
| 5 | 93.8 | 0.319 |
| 6 | 93.9 | 0.317 |
| 7 | 93.8 | 0.316 |
| 8 | 93.9 | 0.315 |
| 9 | 93.8 | 0.313 |
| 10 | 94.0 | 0.310 |

**Supplementary Note 3: Analysis of robust training**

AI D^2^NN operates at the working frequency of 0.85 THz. To ensure the diffractive propagation of the optical field in free space, it’s not recommended for the size of each diffractive neuron more than 0.5 λ. In the main text, we compare the training accuracies of diffractive neural networks with neuron sizes of 100 µm (0.28 λ) and 200 µm (0.56 λ), and confirm that the network with 100 µm neurons exhibits more excellent performance compared to 200 µm counterpart.

Other model parameters are set as follows: the distance from the input plane to the first diffractive layer is set to 1 cm, the spacing between diffractive layers is 0.5 cm, and the distance from the last diffractive layer to the output plane is 1 cm.

In practical experiments, experimental and fabrication errors often lead to significant degradation in model performance. Therefore, robustness training is necessary, which involves introducing random errors such as transverse shifts, rotational misalignments, and displacement errors along the z-axis during the training process. The simulated results of different networks (2 layers to 5 layers) after robust training are shown in Table S2. It can be seen that increasing the number of layers yields only limited improvements in accuracy, while substantially increasing system complexity and interlayer alignment difficulty in experiments, so considering fabrication constraints and alignment difficulty, we selected the 100 µm neuron size and a 2-layer configuration for subsequent experiments.

**Table S2. Comparisons of classification performance across different model settings**

| Models | Training accuracy | Test accuracy |
| --- | --- | --- |
| 100×100 neurons, 2 layers | 93.7% | 87.4% |
| 100×100 neurons, 3 layers | 94.2% | 88.4% |
| 100×100 neurons, 4 layers | 94.3% | 88.6% |
| 100×100 neurons, 5 layers | 94.4% | 88.8% |

Table S3 compares the combined effects of multiple experimental errors on network performance, which are characterized by the test accuracies. It can be seen that as the random errors increase, the performance gap between the network without robustness training and one with robustness training widens. It is evident that robustness training is crucial for the network to resist various errors and maintain good inference performance.

**Table S3. The combined effects of experimental errors on the performance of network**

|  | case | Δ *x* (pixel) | Δ *y* (pixel) | Δ *θ* (rad) | Δ *z* (mm) | w/o robustnesstest accuracy（%） | w/ robustnesstest accuracy（%） |
| --- | --- | --- | --- | --- | --- | --- | --- |
|  | 1 | 0 | 0 | 0 | 0 | 89.3 | 89.3 |
|  | 2 | 1 | 0 | 1 | 0.1 | 84.2 | 88.7 |
|  | 3 | 0 | 1 | 1 | 0.1 | 84.3 | 88.6 |
|  | 4 | 1 | 1 | 1 | 0.1 | 82.1 | 87.4 |
|  | 5 | 2 | 2 | 1 | 0.1 | 71.2 | 83.6 |
|  | 6 | 2 | 2 | 1 | 0.2 | 67.6 | 81.5 |

**Supplementary Note 4: Detailed experimental setup and fabricated samples**

The experimental setup is shown in **Fig. S1**. To ensure precise alignment and spacing between the input mask and two metasurfaces, a high-precision 3D print holder was employed (printer model: JG-A240, fabrication accuracy: 100 μm). During experiments, the stain-less steel mask with an etched input pattern is positioned 10 mm in front of the metasurface to create a predefined spatial intensity distribution. The two metasurfaces are firmly inserted into the holder, maintaining a 5mm spacing. The crystal ZnTe is positioned 10 mm behind the metasurface to capture the transmitted signals. The pump beam passes through the mask and metasurfaces, with incidence on the crystal ZnTe and coincidence with the probe beam. The probe beam is reflected from the left surface of the crystal ZnTe, whose polarization state is modulated by the THz electric field according to the Pockels effect^4^. The probe beam carrying terahertz information exits the crystal ZnTe, and passes through a quarter-wave plate and a Wollaston prism. The CCD (MER-531-20GM-P) acquires the THz time-domain pulse signal via scanning sampling, which is then converted into the THz frequency-domain spectrum through Fourier transformation.

**
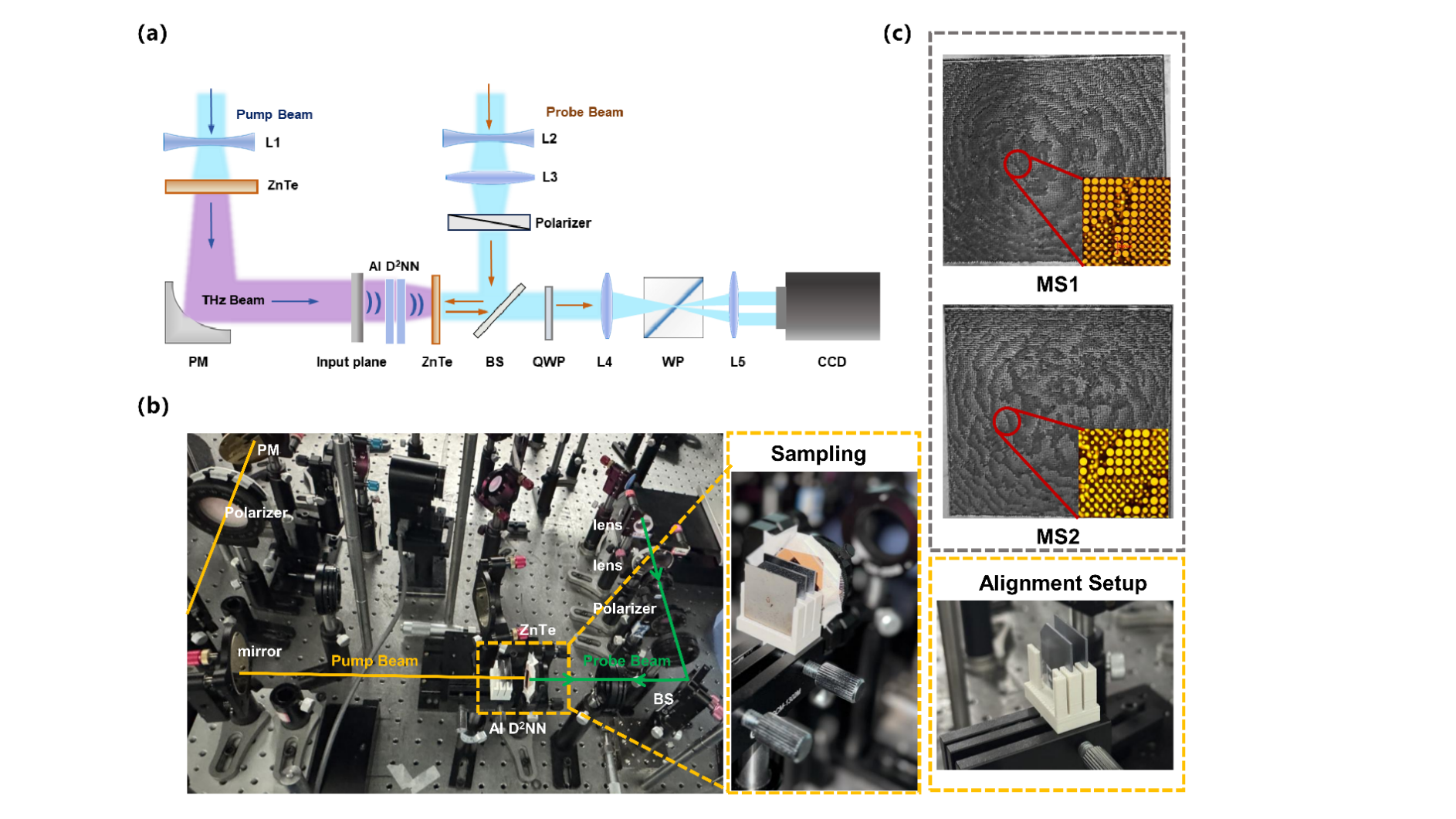
**

**Fig. S1 Experimental setup and fabricated samples. a** The schematic of THz experimental platform. **b** Photograph of the experimental setup. **c** Optical images of the fabricated matasurface. Insets in **c** show the zoom-in microscopic images of meta-atoms.

**Supplementary Note 5: The structural parameters of cylindrical rods**

For ease of fabrication, we selected 16 types of structures whose amplitude coefficients close to 1 and phase responses nearest integer multiples of π/8, and assembled these structures according to the neuron values of the trained diffractive neural network. The parameters of selected cylindrical rods and their corresponding phase coefficients under 0.85 THz (352 µm) are listed in Table S4.

**Table S4. The structural parameters of micropillar structures**

| Label | 1 | 2 | 3 | 4 | 5 | 6 | 7 | 8 |
| --- | --- | --- | --- | --- | --- | --- | --- | --- |
| Radius (µm) | 15 | 20 | 23 | 25 | 28 | 30 | 32 | 33 |
| Label | 9 | 10 | 11 | 12 | 13 | 14 | 15 | 16 |
| Radius (µm) | 35 | 36 | 37 | 38 | 39 | 41 | 42 | 43 |

**Supplementary Note 6: Energy distribution for the AI D^2^NN**

Analyzing the electric field distribution at the output plane is crucial for assessing the network’s capability to recognize targets 0-5 in multi-object scenes. In addition, other evaluation metrics such as energy efficiency *E*, normalized energy contrast *ΔE* and signal-to-noise ratio *SNR*, must be considered to ensure that the classification results are distinct and reliably represented.

First, the energy distribution across 6 classification regions is calculated as follows:

$\left[\begin{matrix} P_{0}=\sum i_{1}\leq i\leq i_{2}\sum j_{1}\leq j\leq j_{2}|E_{(i,j)}|^{2} \\ P_{1}=\sum i_{3}\leq i\leq i_{4}\sum j_{3}\leq j\leq j_{4}|E_{(i,j)}|^{2} \\ P_{2}=\sum i_{5}\leq i\leq i_{6}\sum j_{5}\leq j\leq j_{6}|E_{(i,j)}|^{2} \\ P_{3}=\sum i_{7}\leq i\leq i_{8}\sum j_{7}\leq j\leq j_{8}|E_{(i,j)}|^{2} \\ P_{4}=\sum i_{9}\leq i\leq i_{10}\sum j_{9}\leq j\leq j_{10}|E_{(i,j)}|^{2} \\ P_{5}=\sum i_{11}\leq i\leq i_{12}\sum j_{11}\leq j\leq j_{12}|E_{(i,j)}|^{2} \end{matrix}\right]$ (S7)

In each row, i and j denote the starting and ending positions of the column and row coordinates in the classification regions, respectively.

In this work, the classifier is designed for six-class classification, the total energy in classification regions, output plane and input plane is calculated separately, and the calculation process is as follows.

$P_{\text{total}}=P_{0}+P_{1}+P_{2}+P_{3}+P_{4}+P_{5}$ (S8)

(S9)

(S10)

The energy efficiency for the maximum energy among six regions is calculated as:

$E=\frac{P_{\max}}{P_{\text{total}}}$ (S11)

The normalized energy contrast ΔE represents the ratio of the difference between the highest and second-highest energies across the six detection regions to the highest energy, which can be expressed as:

$\Delta E=\frac{P_{\max}-P_{\text{second}}}{P_{\max}}$ (S12)

The signal-to-noise ratio *SNR* here is defined as the ratio of the maximum average intensity of the classification regions to the average intensity of all other areas on the output plane excluding the six detection regions, the mathematical expression is as follows,

$SNR=\frac{P_{\max}/49}{(P_{\text{out}}-P_{\text{total}})/(10000-294)}$ (S13)

Here, 10000 represents the total pixel count on the output plane, while 49 denotes the number of pixels occupied by a single classification region, and six classification regions totally occupy 294 pixels.

**Supplementary Note 7: Trained network’s classification results of different cases**

This section provides further details about the experimental results, specifically supplementing the recognition performance analysis of the trained AI D^2^NN for handwritten digits classification in multi-object scenarios.


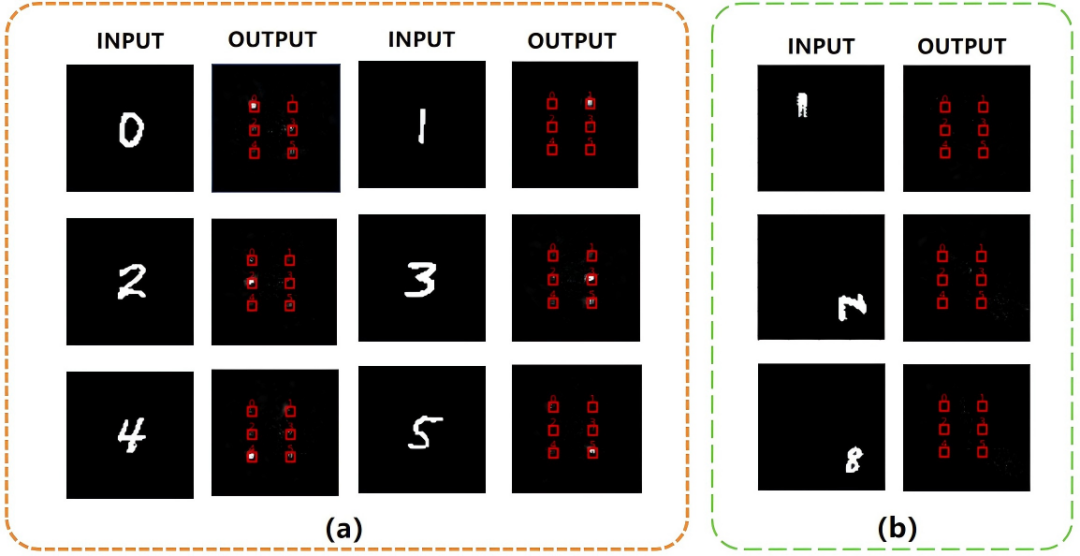


**Fig. S2 Trained network’s classification results of different objects. a** Simulation results of classifying single target **b** Simulation results of classifying single interference

First, the optical field distributions of the network recognizing a single digit (0–5) or an interfering object are illustrated in **Fig. S2.**

Next, the AI D^2^NN demonstrates feasibility in multi-object scenes. Here, “multi-object” refers to a scene containing multiple objects, where the network can classify the primary target without being influenced by other interfering objects. First, the classification performance in scenes with targets and various types of interference, including intra-class, inter-class, and dynamic interference, is presented in **Fig. S3(a)–(d)**, respectively.


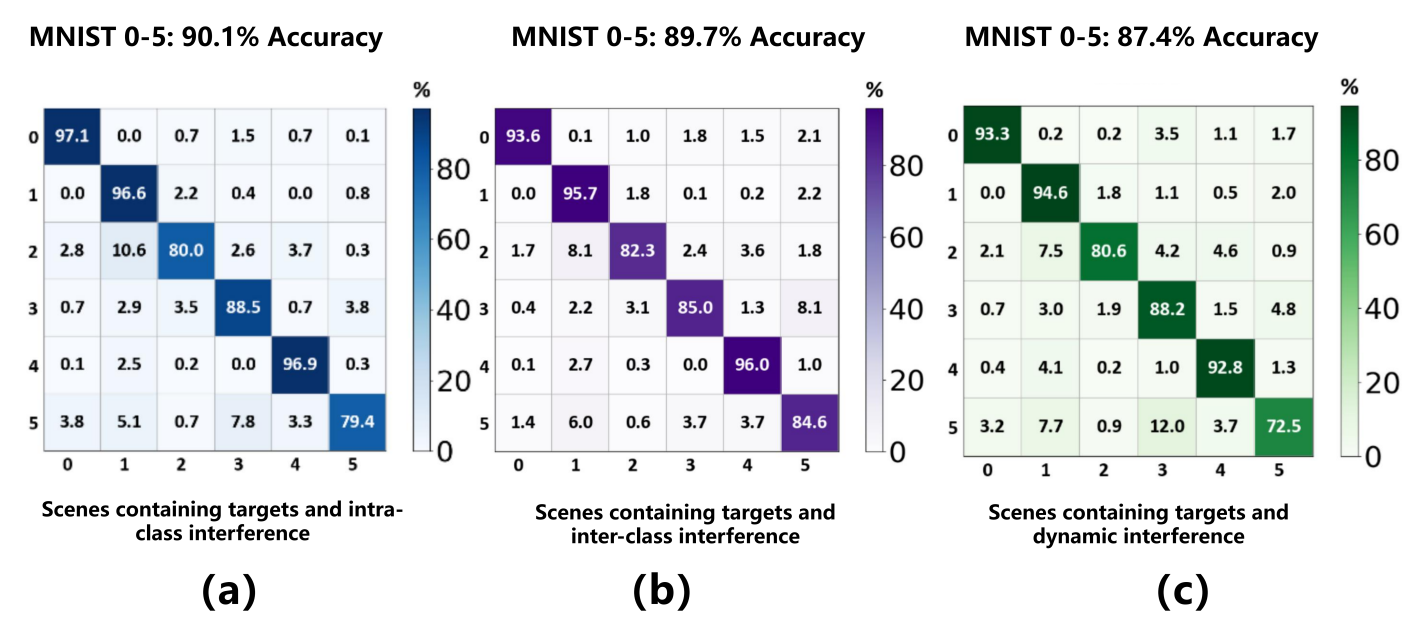
**Fig. S3 The recognition accuracies and confusion matrices of the AI D2NN for the three types of interference. a** Intra-class interference. **b** Inter-class interference. **c** Dynamic interference.

Besides, the AI D^2^NN demonstrates the capability to recognize handwritten digits 0-5 in dynamic multi-object scenes. Subsequently, we randomly tested scenarios containing intra -class interference, inter-class interference, and dynamic interference. The results of network classification performance for digits 0-5 are shown in **Fig. S4**.

**Fig. S4** shows that discrepancies exist between the simulation and experimental results, which mainly arise from multiple error sources, including fabrication imperfections, light source instability, and detection module inaccuracies. These issues can be mitigated by improving the exposure resolution in nanofabrication, employing high-precision alignment platforms, stabilizing the light source in polarization and intensity, and reducing the scanning step size or increasing repeated measurements during detection.


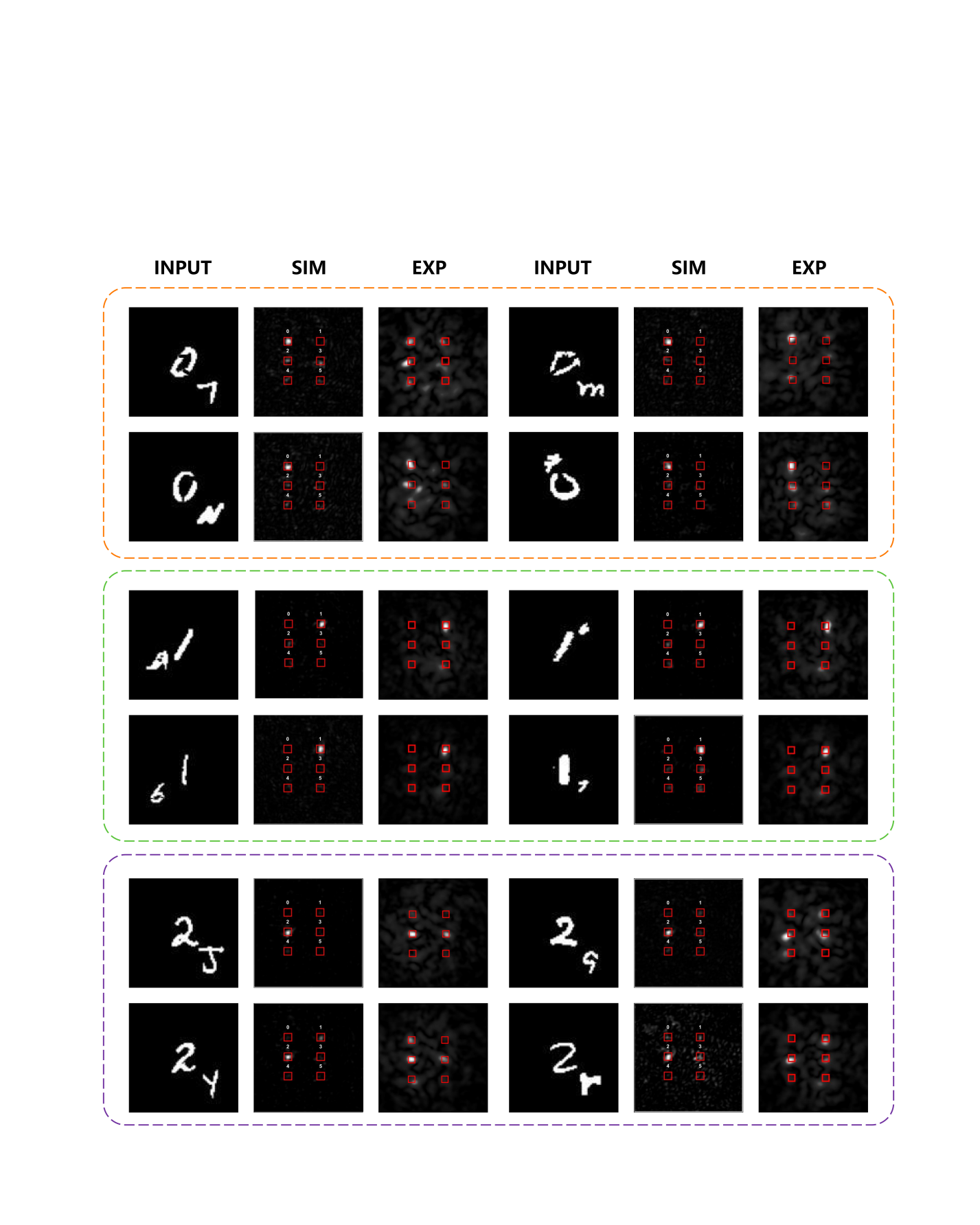


**(Continued on next page)**


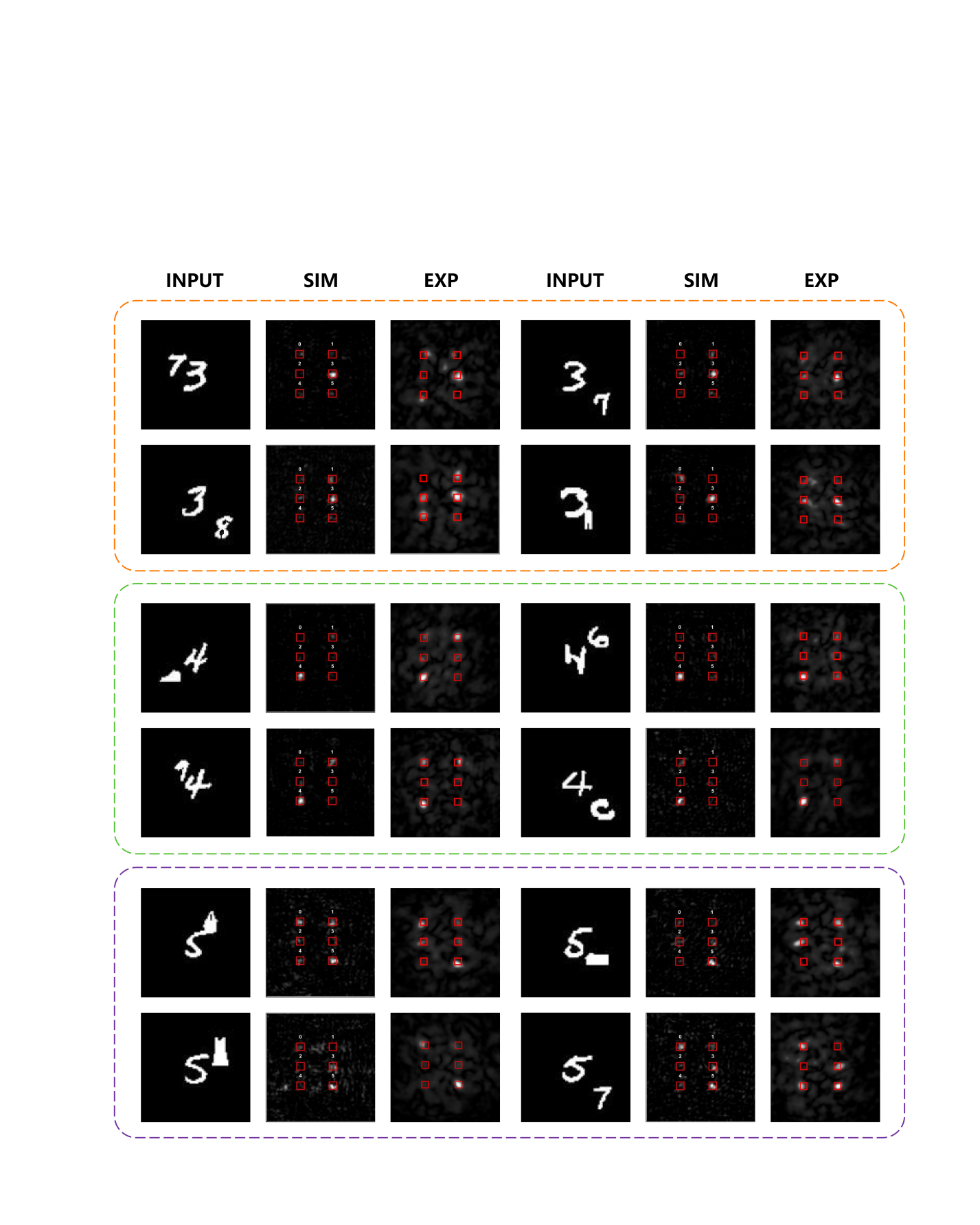


Fig. S4 Energy distribution of numerical and experimental results for the classification of digits 0-5 in multi-object scenes.

Optical neural networks can perform computations at the speed of light. Therefore, the AI D^2^NN is capable of recognizing handwritten digits in scenes containing dynamic interference. The simulation results are presented in **Fig. S5**.


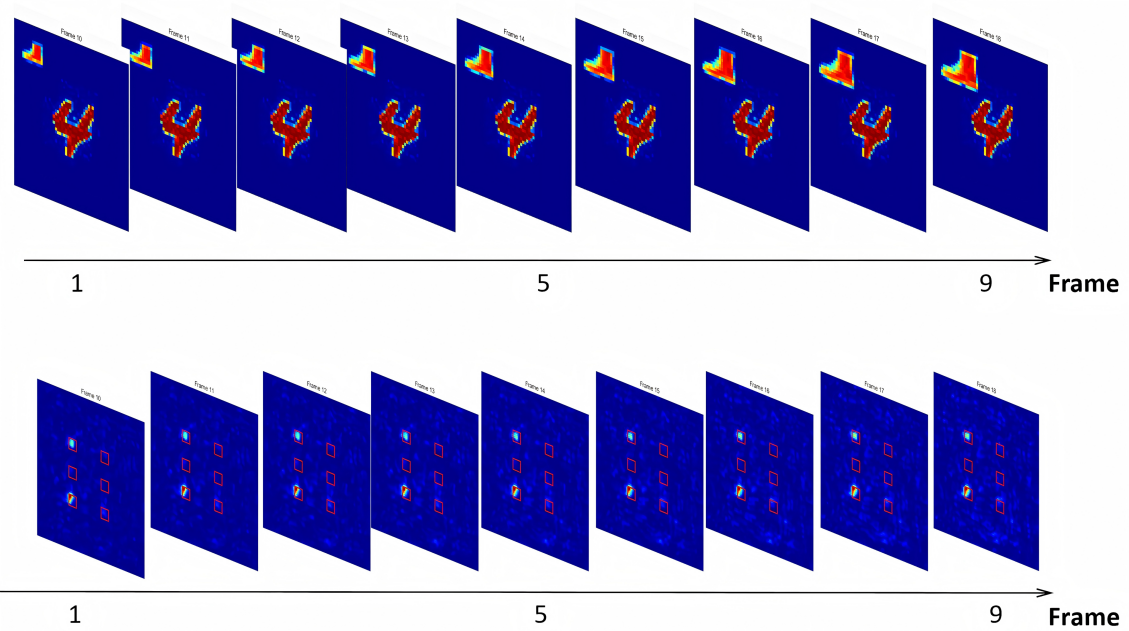


**Fig. S5 Energy distribution of numerical results for the classification of digits 0-5 in scenes containing dynamic interference.**


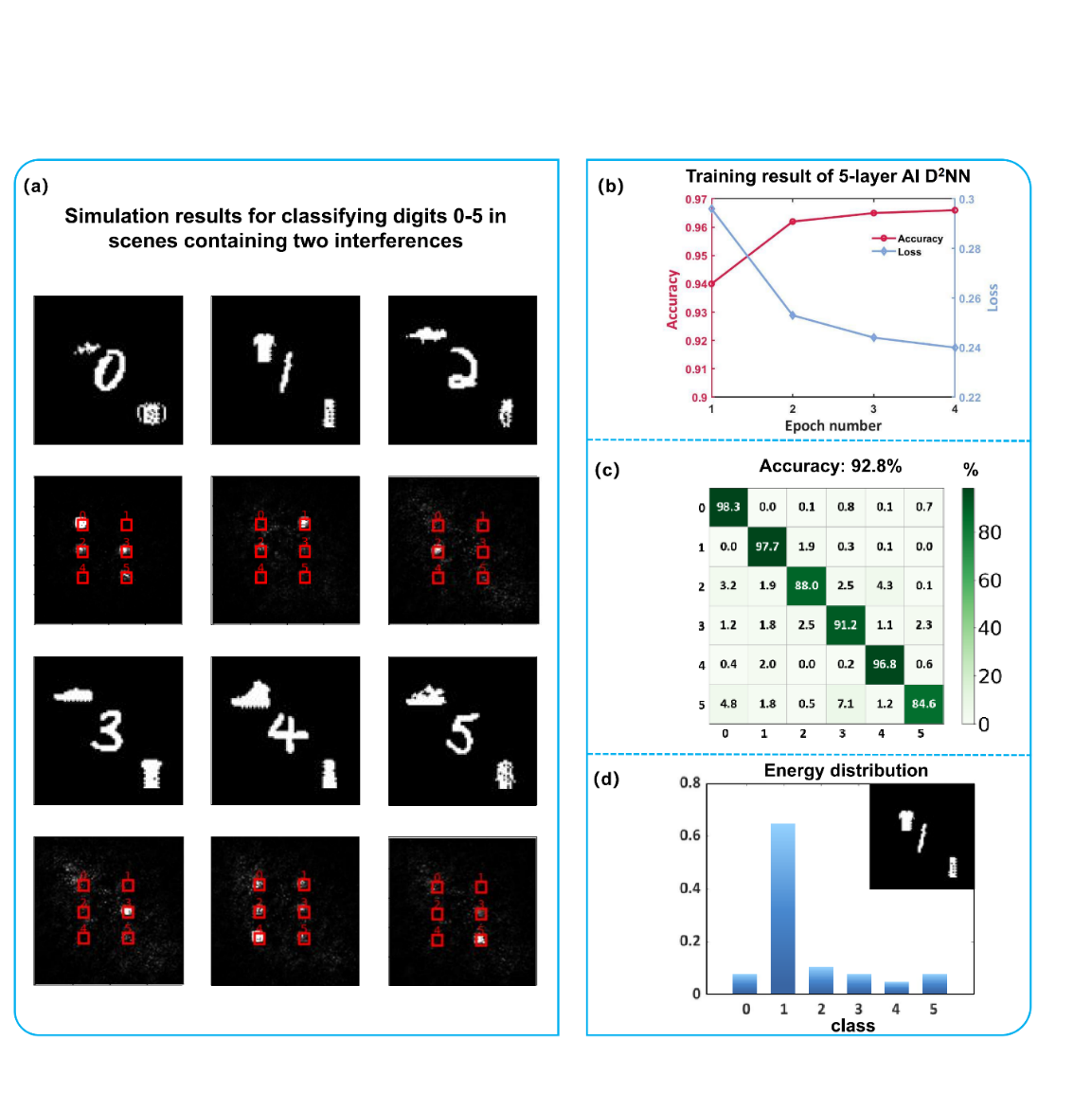


**Fig. S6 Simulation results for classifying digits 0-5 in scenes containing two interferences.** **a** The output plane’s energy distribution. **b** Training result. **c** Confusion matrix of test dataset and test accuracy. **d** Specific energy distribution when scenes containing digit 1, trousers and T-shirt.


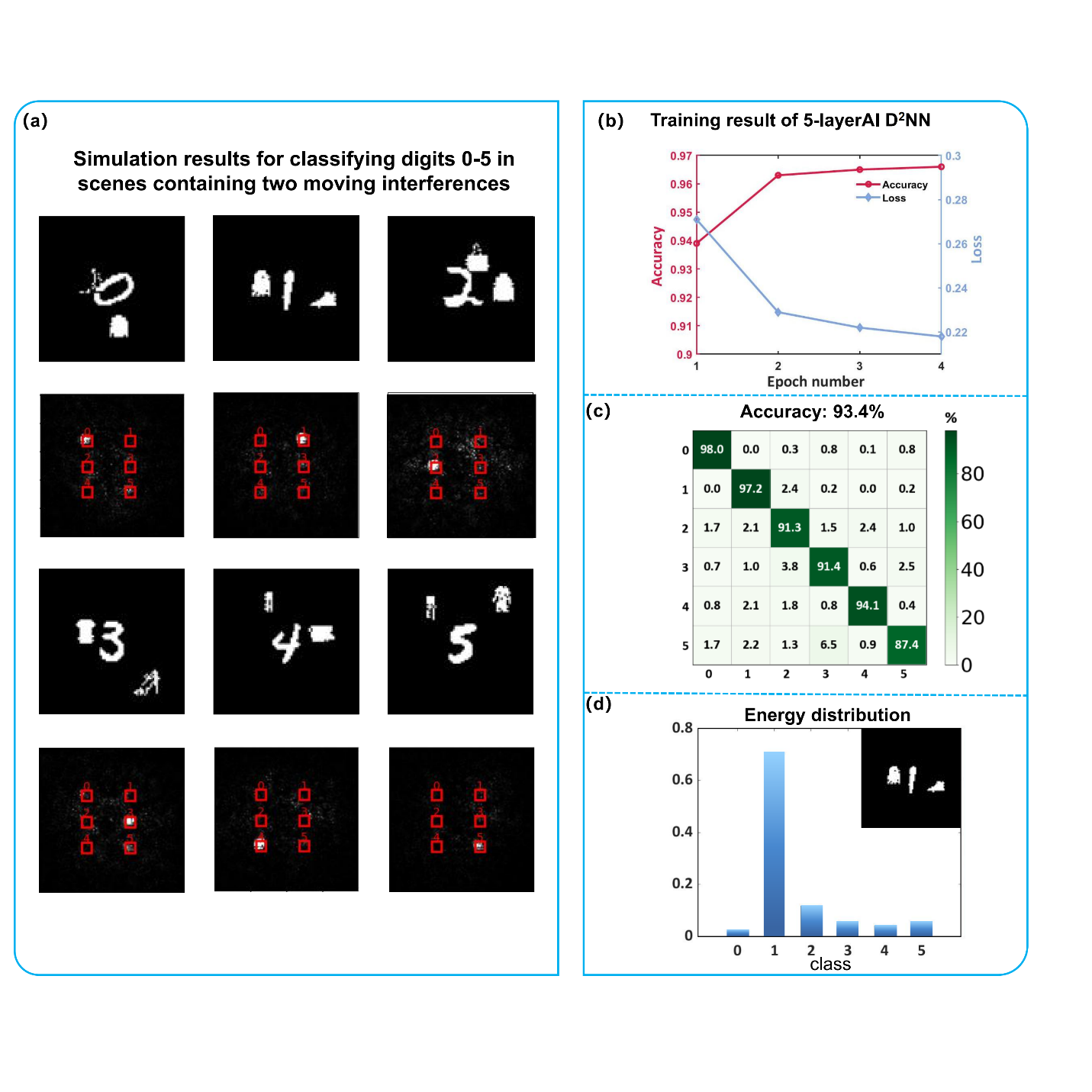


**Fig. S7. Simulation results for classifying digits 0-5 in scenes containing two moving interferences.** **a** The output plane’s energy distribution. **b** Training result. **c** Confusion matrix of test dataset. **d** Specific energy distribution when scenes containing digit 1, coat and shoes.

The concept of transforming the optical fields of non-target objects into background noise through ONN is inherently extendable, for example, even as the number and categories of interference increase, the network still demonstrates excellent capability in classifying the primary target in multi-object scenarios. In theory, more complex scenarios for target recognition can be achieved by employing more diverse and extensive datasets, more rigorous loss functions and deeper network models during training phase.

Therefore, we constructed a 5-layer AI D^2^NN to adapt to scenarios featuring a single target and more than two interfering objects, where the positions and sizes of all interfering objects vary flexibly. The simulation results for classifying digits 0-5 in scenes containing two interfering objects are shown in **Fig. S6**. The simulation results for classifying 0-5 in scenes containing two moving interfering objects are presented in **Fig. S7**.


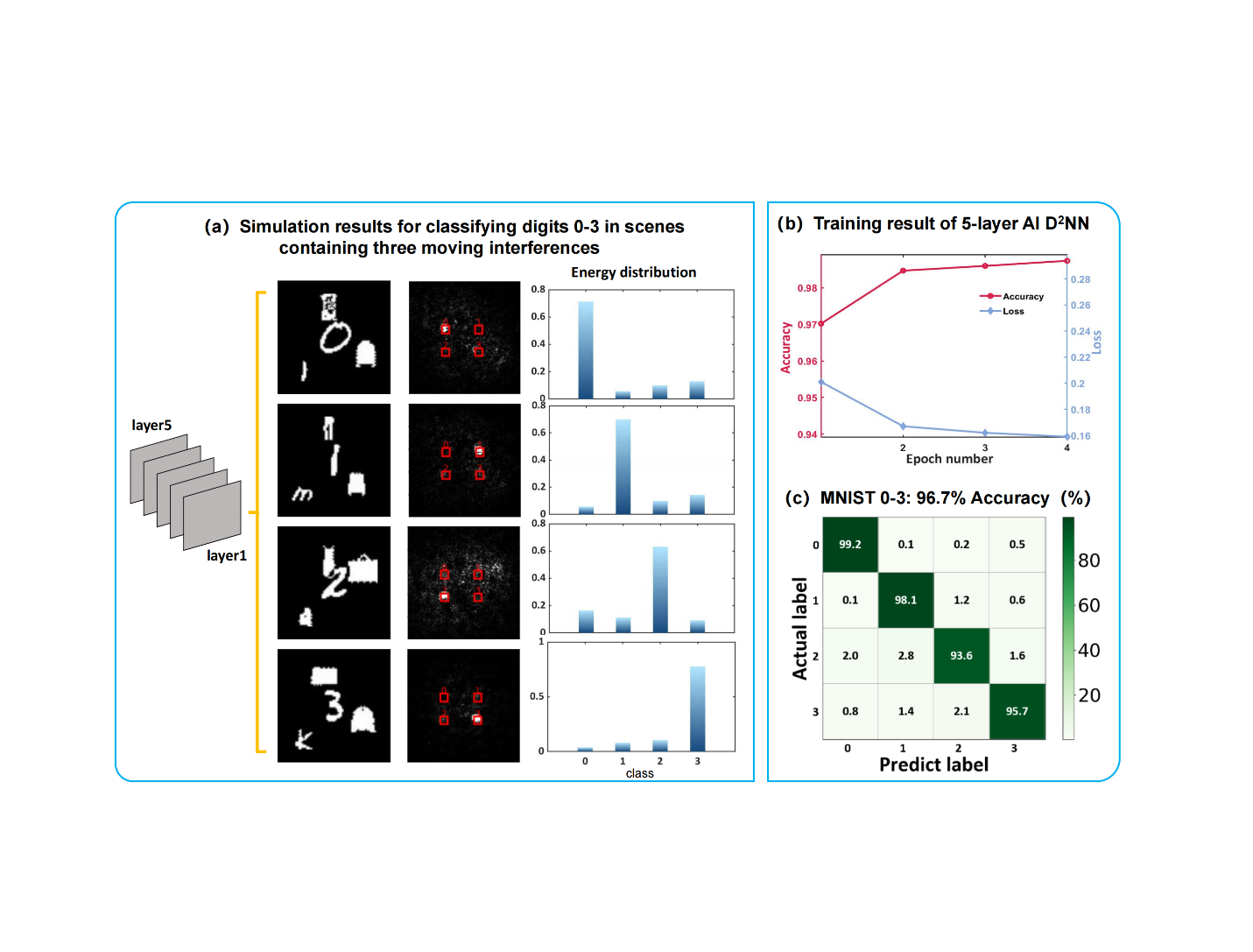


**Fig. S8. Simulation results for classifying digits 0-3 in scenes containing three moving interferences. a** The output plane’s energy distribution. **b** Training result. **c** Confusion matrix of test dataset.


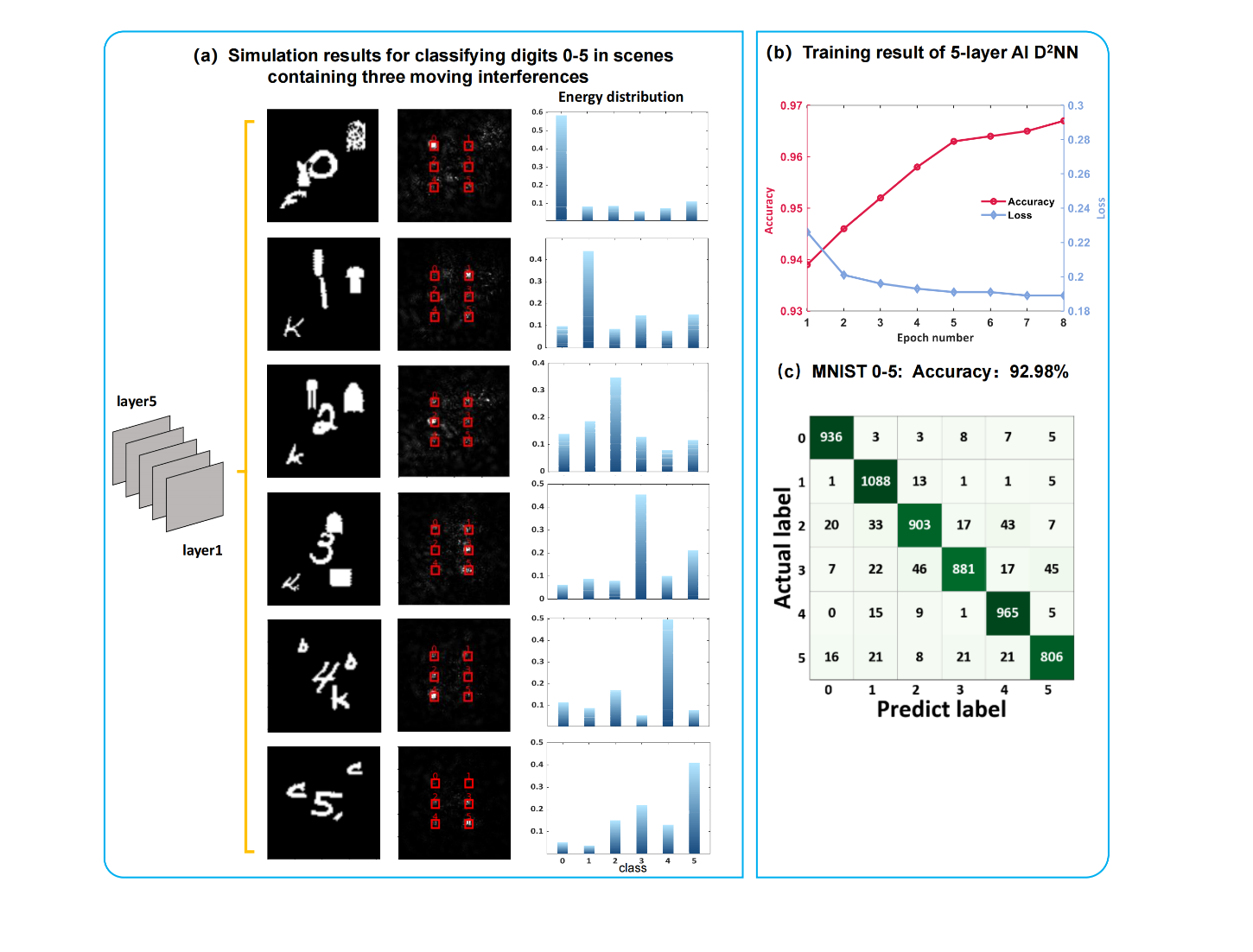


**Fig. S9. Simulation results for classifying digits 0-5 in scenes containing three moving interferences. a** The output plane’s energy distribution. **b** Training result. **c** Confusion matrix of test dataset.

The simulation results for classifying 0-3 in scenes containing 3 moving interfering objects are illustrated in **Fig. S8**. The simulation results for classifying 0-5 in scenes containing 3 moving interfering objects are illustrated in **Fig. S9**. Above results manifest that the AI D^2^NN has great potential for implementing complex tasks.

Apart from digit classification, the AI D^2^NN can be further extended to recognize a larger number of categories and more complex objects. Therefore, a more advanced model consisting of 3 diffractive layers with 10,000 neurons each is constructed to handle the 10-class classification of handwritten digits (MNIST), the six-class classification of fashion items (Fashion-MNIST), and graffiti objects (QuickDraw! dataset). The simulation results for these three tasks are presented in **Figs. S10-S12**.


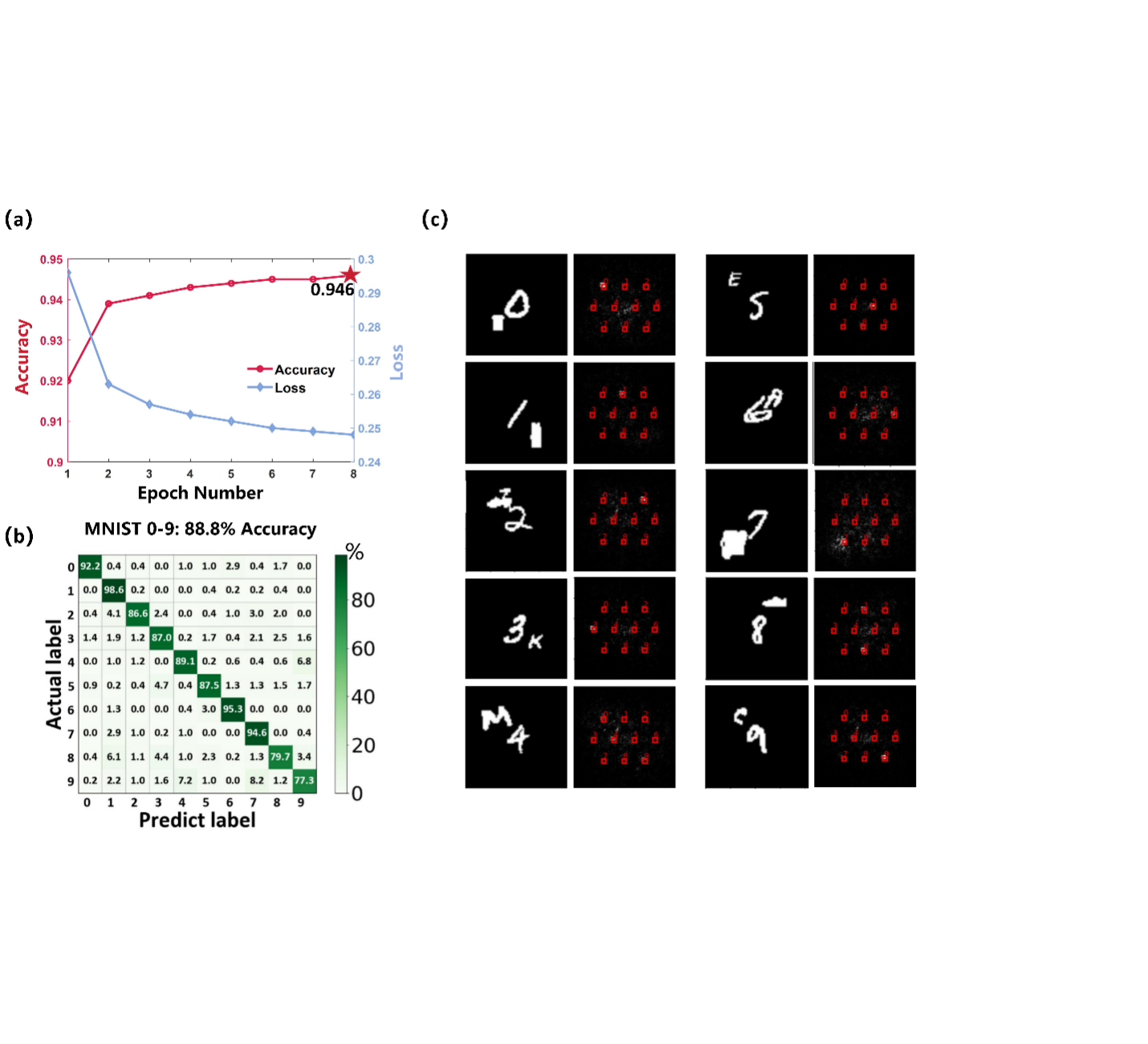


**Fig. S10. Simulation results of 10-class classification of digits under multi-object scenes.** **a** Training result. **b** Confusion matrix of test dataset. **c** The energy distribution of simulation output.

**
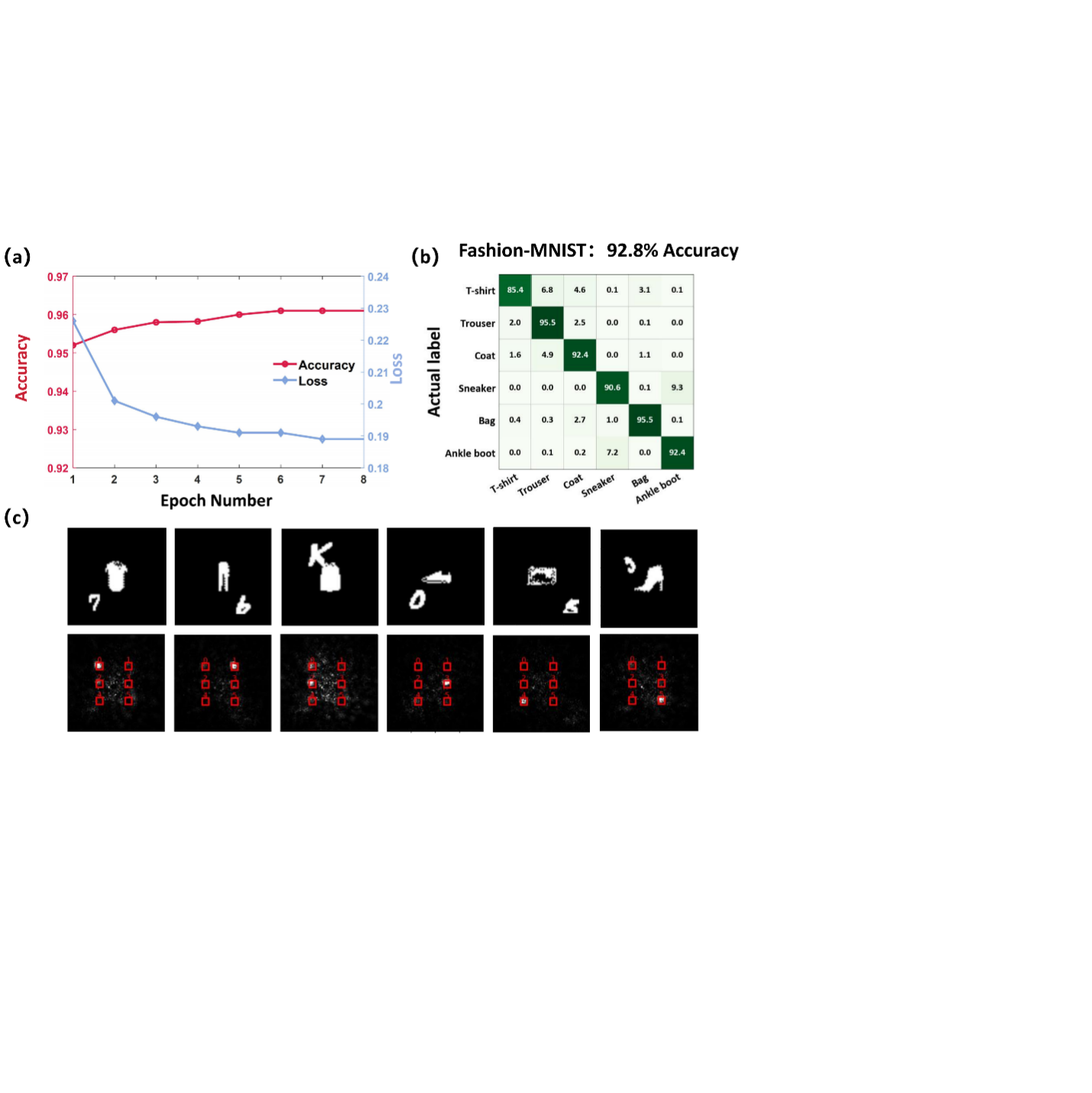
**

**Fig. S11. Simulation results of 6-class classification of fashion items under multi-object scenes. a** Training result. **b** Confusion matrix of test dataset. **c** The energy distribution of simulation output.

**
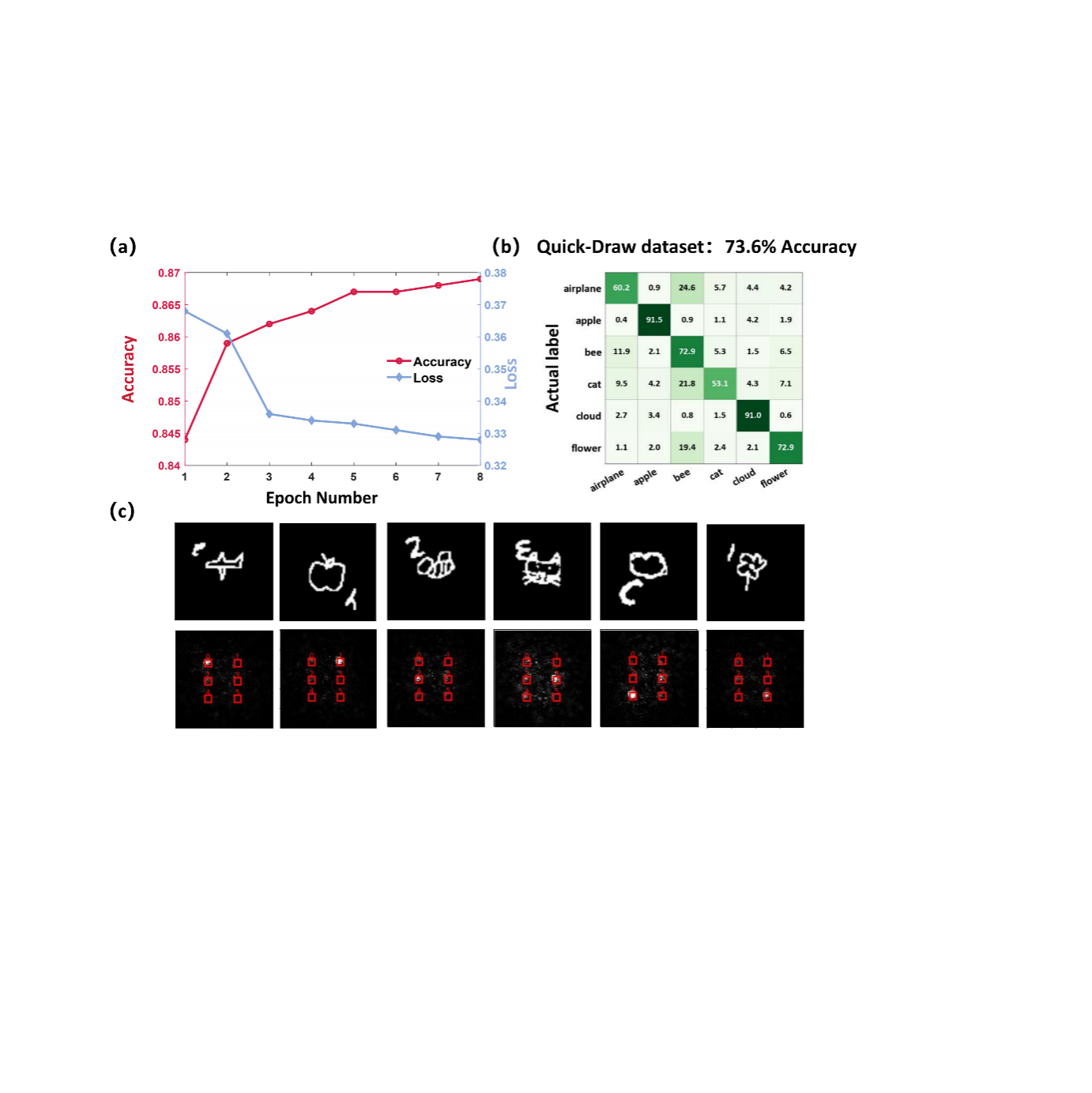
**

**Fig. S12. Simulation results of 6-class classification of graffiti objects under multi-object scenes. a** Training result. **b** Confusion matrix of test dataset. **c** The output plane’s energy distribution.

**Supplementary Note 8: Analysis of the Effect of Different Interference on Network’s Classification Performance**

The size and shape of the interference object can affect the network’s performance. To investigate this, we conduct the following simulations to analyze the test accuracy of the network under different size ratios of target-to- interference, as well as different shape and category of interference, as shown in **Fig. S13.**

**Fig. S13 shows the influence of interference size, shape, and category on the classification** accuracy of the network. As illustrated in the left panel, when the size ratio of interference to target increases from 0.2-0.5 to 1.0-2.0, the test accuracy decreases from 89.7% to 74.7%, indicating that larger interference has a negative impact on recognition performance. The middle panel compares the results between solid-shaped and line-shaped interference, revealing similar levels of accuracy (87.0% vs. 88.3%), suggesting that the interference shape has a relatively minor effect. The right panel presents the results when interference is drawn from different datasets, including MNIST 6-9, Fashion-MNIST, EMNIST, and QuickDraw! dataset. The classification accuracy remains high (around 90%) across all interference types, demonstrating the robustness of the network to various visual features.

**
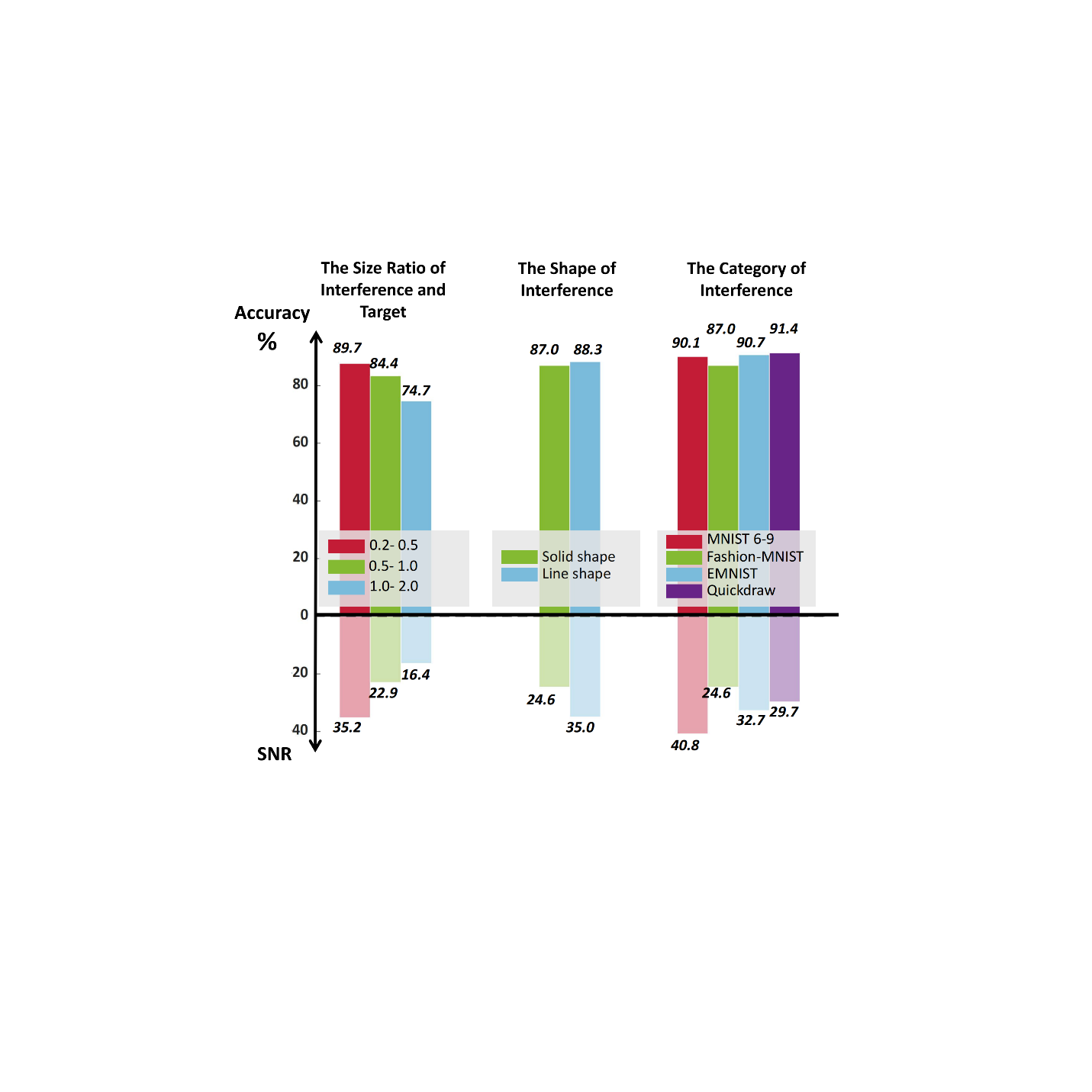
**

**Fig. S13. The impact of different interference on network’s performance**

Additionally, the contrast between targets and interfering objects plays a role in the correct identification of the targets. Thus, we redesigned the testing dataset by assigning different grayscale values to the target and interference objects in the test images, thereby simulating scenes containing target and interference with various brightness contrasts. A comparative analysis of recognition accuracy and signal-to-noise ratio *(SNR)* under different target-to-interference brightness ratios was conducted using the same AI D^2^NN model. The final results are shown in **Table. S5.**

**Table. S5. The comparison of classification performance with different brightness ratios**

| **B(Target) / B(Interference)** | **Test accuracy (%)** | **Signal-to-Noise Ratio *(SNR)*** |
| --- | --- | --- |
| 0.5 | 87.3 | 23 |
| 0.7 | 87.4 | 29 |
| 1.0 | 87.4 | 31 |
| 1.25 | 90.1 | 37 |
| 2.0 | 90.1 | 39 |

According to the simulation results, when the brightness ratio of target and interference is less than 1, the network’s test accuracy remains almost unaffected, while the *SNR* exhibits a linear decline as the brightness contrast decreases. When the brightness ratio exceeds 1, both the test accuracy and *SNR* increase, as more target’s intensity concentrating in the designated region. This enhances the contrast between the focused spot and the background noise on the output plane, thereby improving the model’s robustness to interference and increasing the probability of correct classification. Therefore, the impact of different brightness on the classification accuracy is almost negligible.


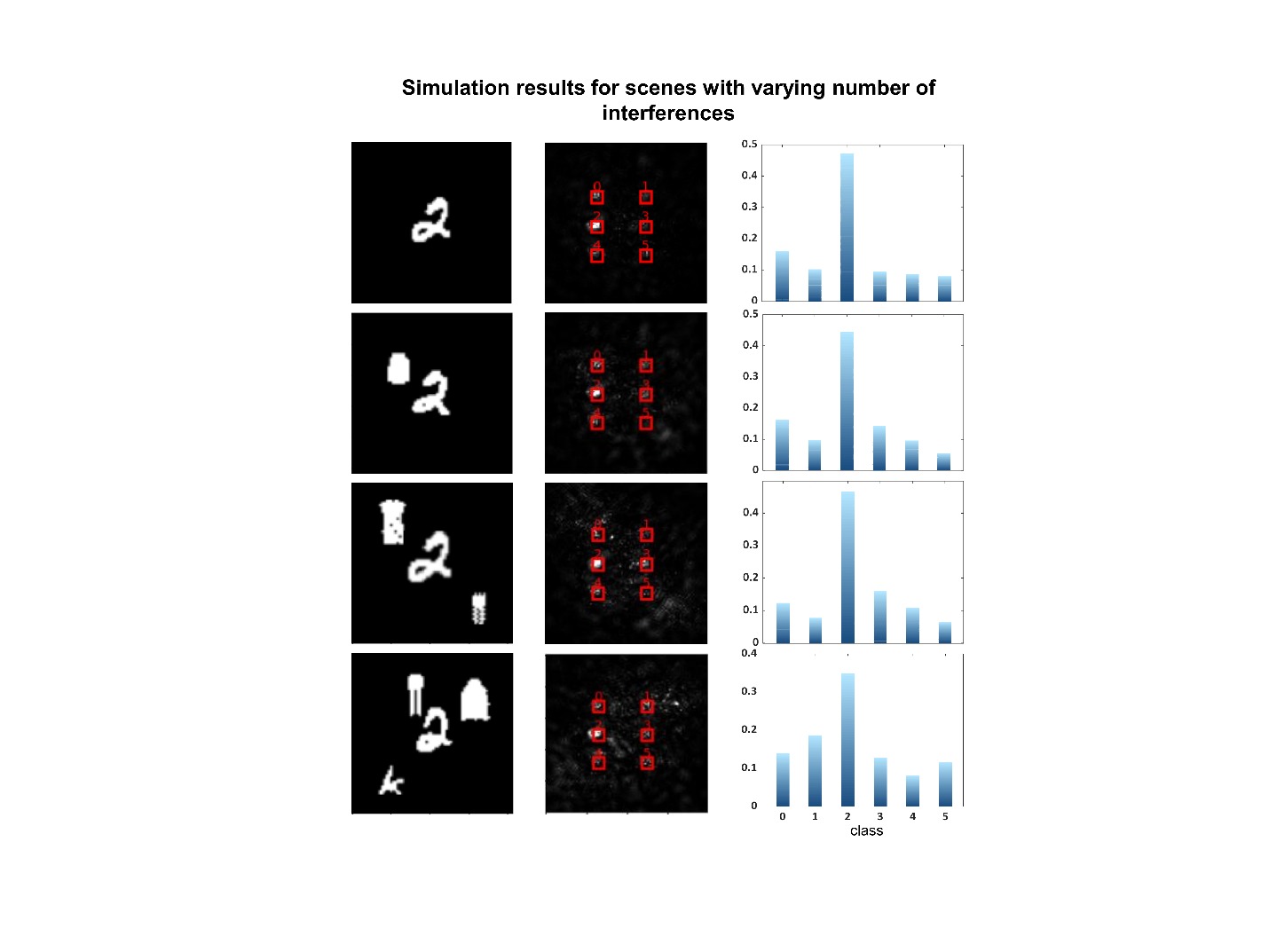


**Fig. S14. Simulation results for scenes with varying numbers of objects**

Last but not least, the number of interfering objects may influence the classification performance of network, since additional interfering object may increase the crosstalk between 6 detection windows. Thus, we compared the classification performance of the same D^2^NN model under several test scenarios. The test scenarios included: target only, one target with an interfering object, one target with two interfering objects, and one target with three interfering objects. The optical field distributions are shown in **Fig. S14**. Meanwhile, we calculated the average energy distribution within 6 detection windows, based on 100 input images from different classes (digit 0-5), as presented in **Figs. S15**-**S17**.

According to the simulation results, the classification performance of the network remains stable when the number of interference objects in the scene is fewer than 3. When 3 interference objects are introduced, however, a noticeable decrease occurs in the energy proportion focused within the correct detection window. This degradation is likely caused by variations in the size and shape of the interference objects, which misleads the networks to redistribute the optical field on the output plane and generate faint light spots in other detection regions.

According to the statistical results of 100 samples each category, a sum of 600 samples for each test. Overall, the energy distribution curves of categories “2”, “4”, “5”, and “6” maintain consistent trends across different interference conditions, indicating that the network can still concentrate most of the optical energy in the correct detection region despite the presence of interference. In contrast, the recognition results for categories “0” and “1” exhibit less stability. The energy contrast between the correct and incorrect detection regions becomes less distinct. This instability is mainly attributed to the partial occlusion of key contour features by the interference objects, and the probability of such occlusion increases with the number of interfering sources.

**
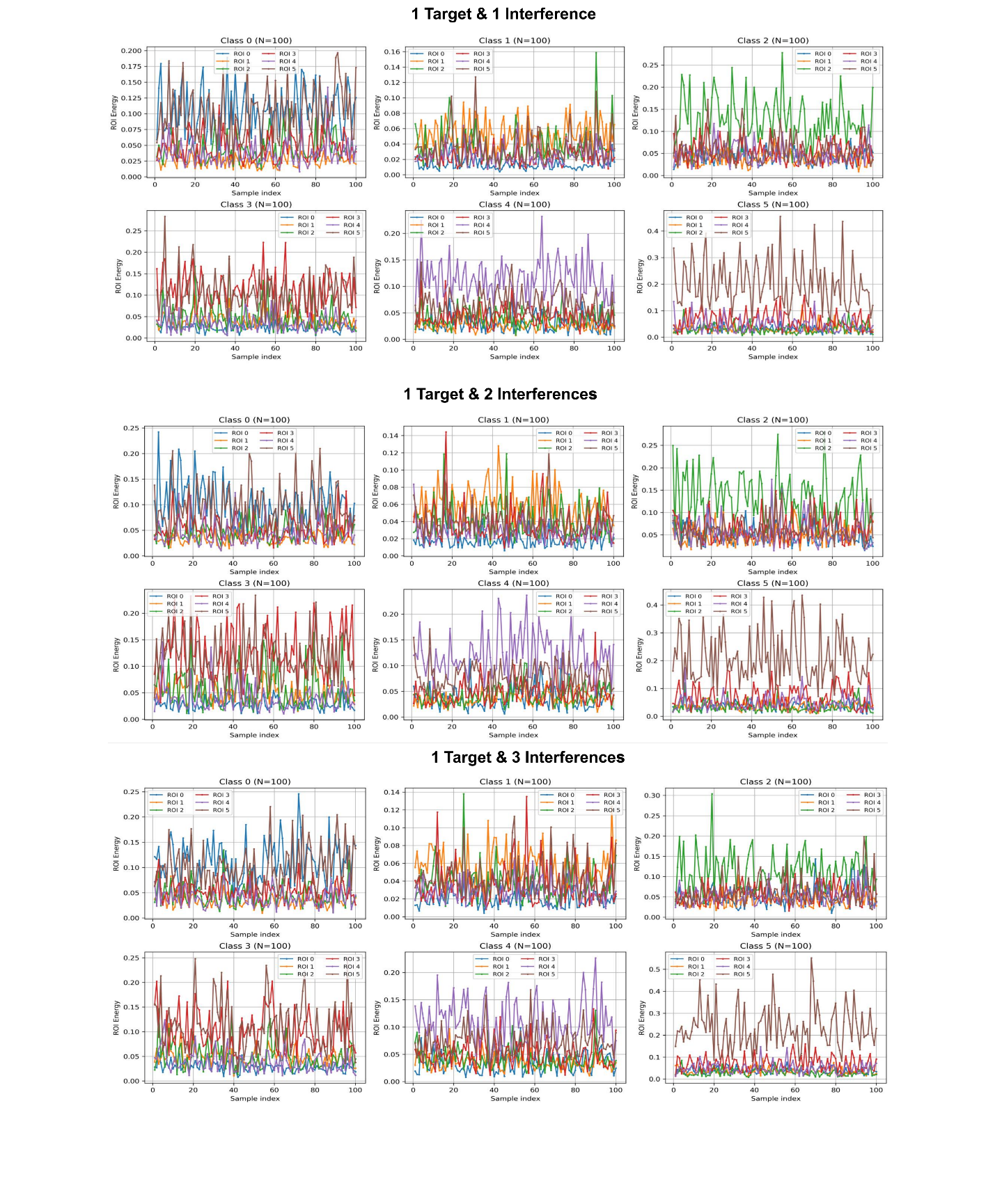
**

**Fig. S15. Statistical analysis of the model’s classification performance in scenes with a target and an interference.** A total of 600 tests were conducted, with each digit evaluated 100 times (represented by each grid). The figure illustrates the energy distribution across six detection regions.

**
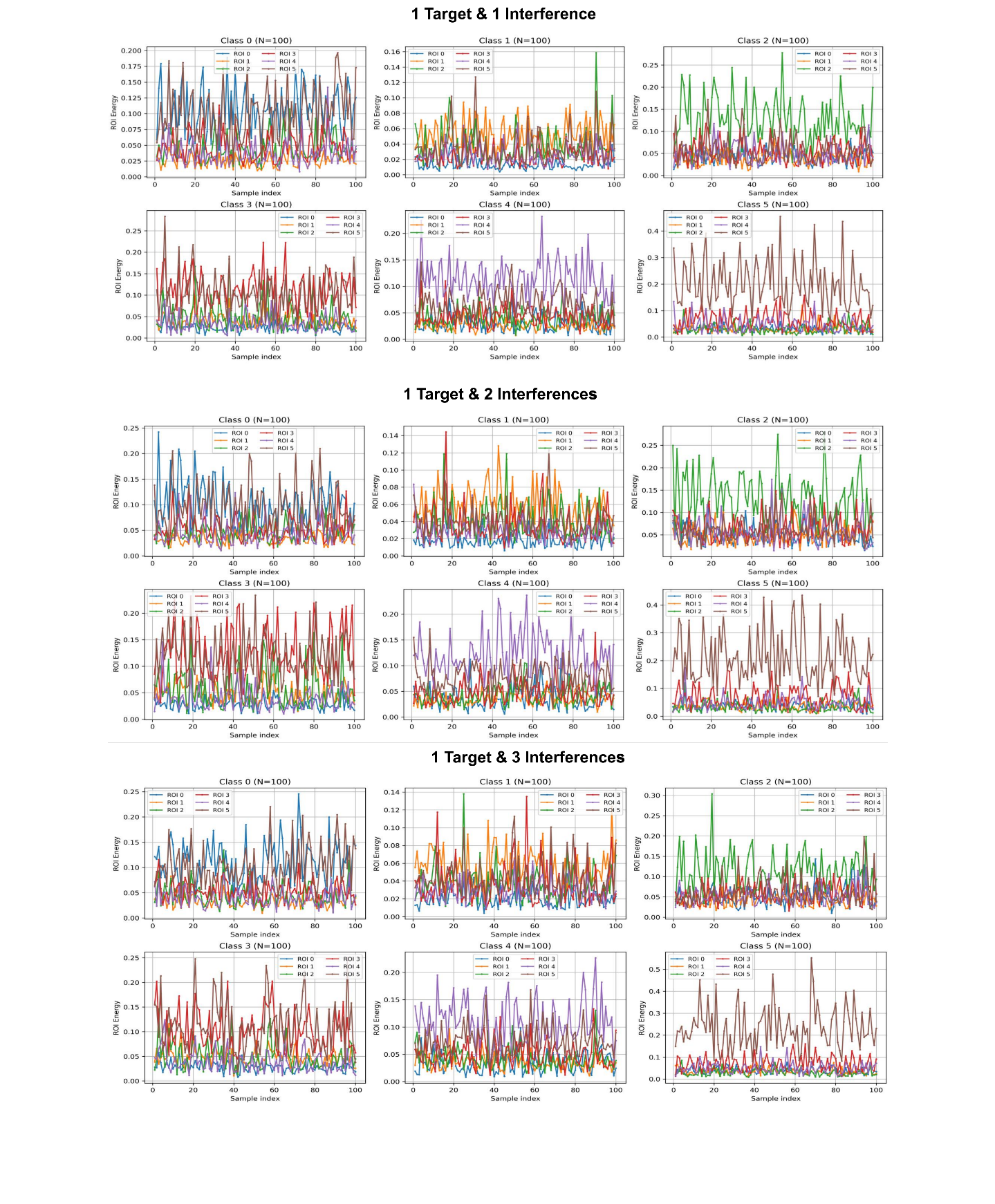
**

**Fig. S16. Statistical analysis of the model’s classification performance in scenes with a target and 2 interferences.** A total of 600 tests were conducted, with each digit evaluated 100 times (represented by each grid). The figure illustrates the energy distribution across six detection regions.

**
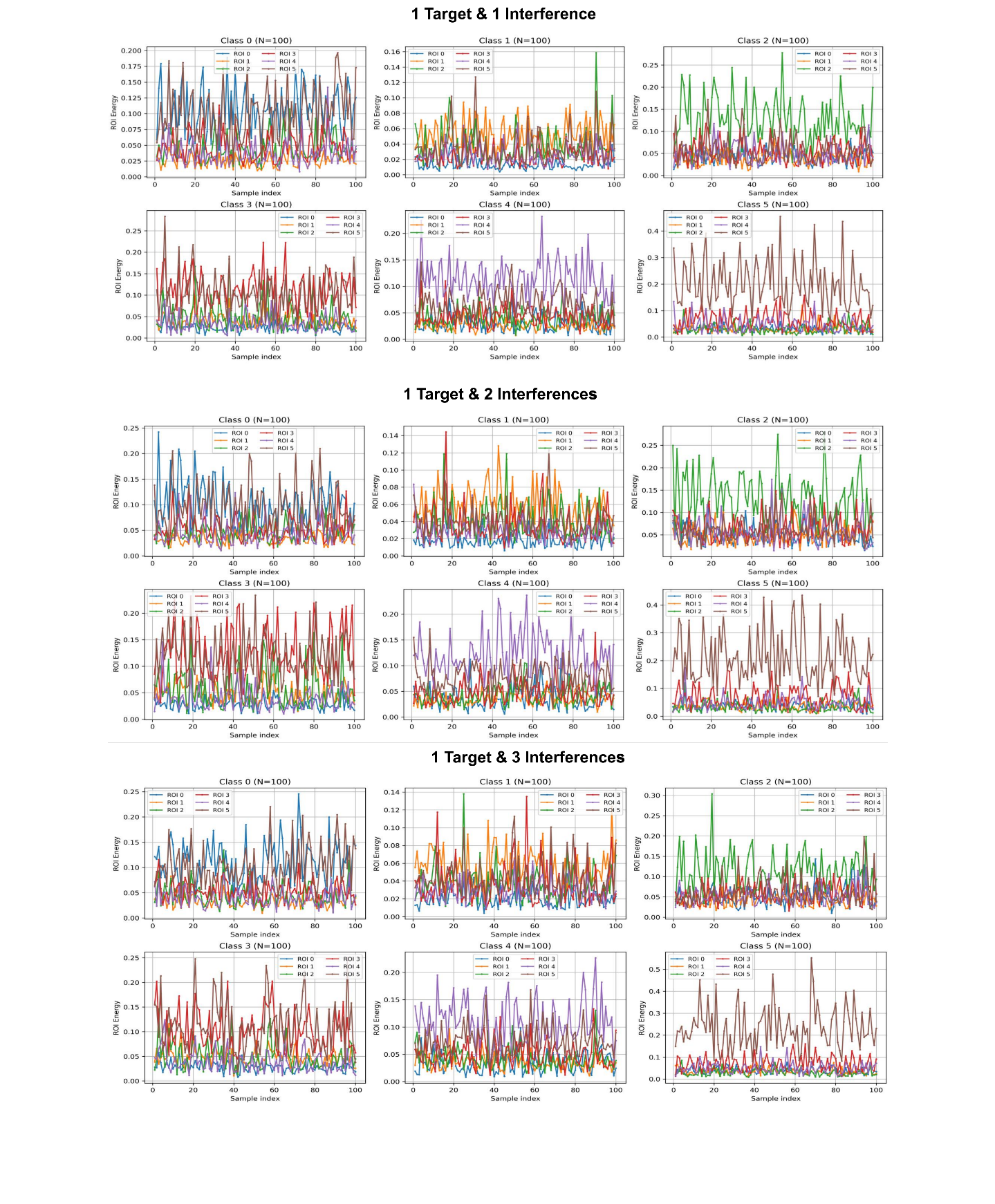
**

**Fig. S17. Statistical analysis of the model’s classification performance in scenes with a target and 3 interferences.** A total of 600 tests were conducted, with each digit evaluated 100 times (represented by each grid). The figure illustrates the energy distribution across six detection regions.

**Supplementary Note 9: The improvement for network’s classification performance**

To improve the network’s classification performance, we fine-tune the network’s parameter settings and optimize the modules, including adjustments such as fine-tuning interlayer spacing, increasing the number of neurons and diffractive layers, and introducing nonlinear functions, as shown in **Fig. S18 (a)** to **Fig. S18 (d)**^5, 6, 7, 8^.

As shown in **Fig. S18**, by fine-tuning the model parameters-such as increasing the number of neurons to 200×200, adding more diffractive layers to 4, or introducing a nonlinear structure—the recognition accuracy of the network can exceed 96%. This improvement indicates that, the input–output mapping relationship can be more effectively fitted with more parameters, leading to superior overall performance of the model.

**
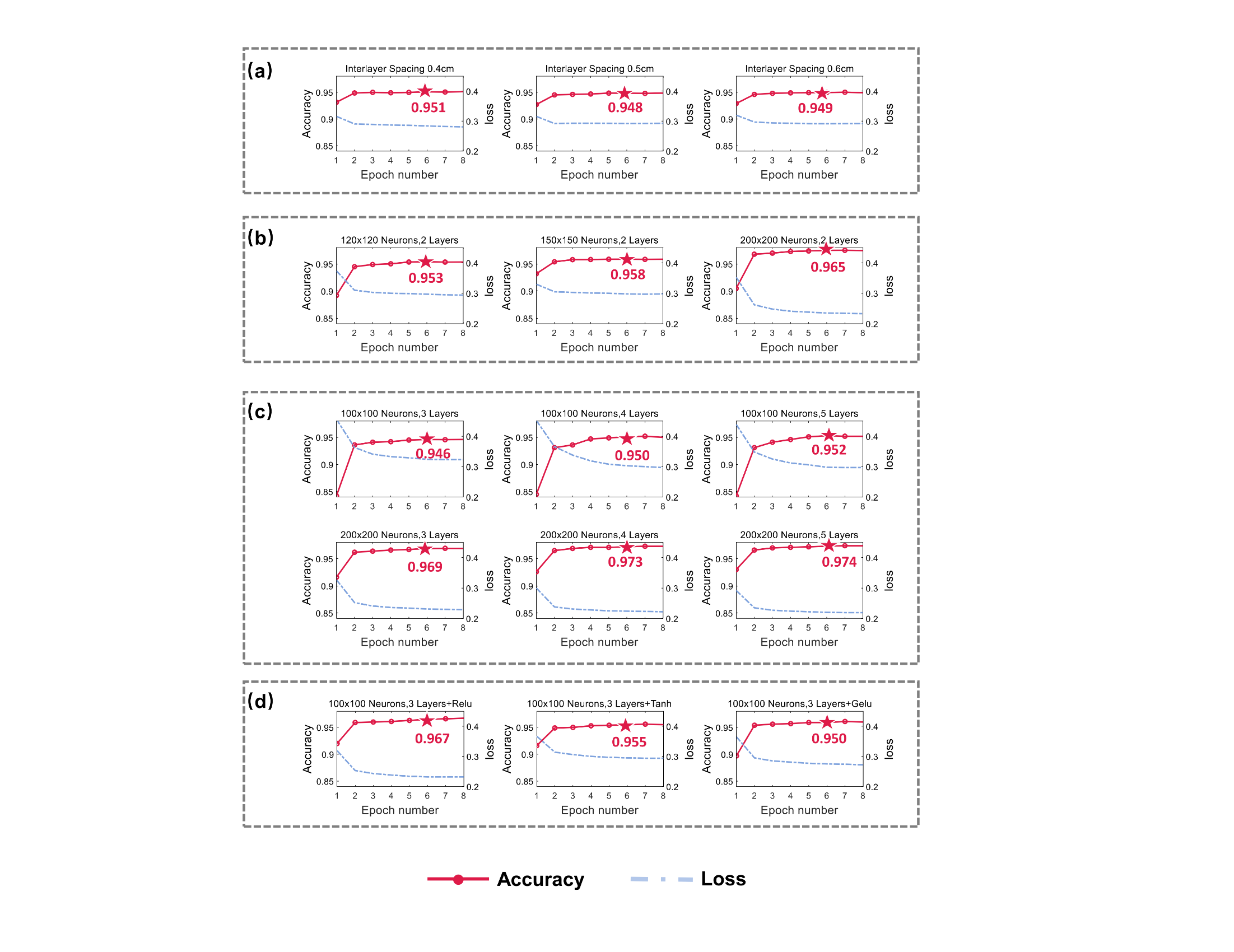
**

**Fig. S18. The simulation validations for specific modifications of the networks. a** Fine-tuning interlayer spacing. **b** Dual layer network with increased neurons each layer. **c** Increasing diffractive layers with 100×100 and 200×200 neurons each layer. **d** Introducing different nonlinear functions commonly used in ENNs.

In future work, the AI D^2^NN can achieve multi-object recognition by integrating multi-dimensional optical encoding technology, as shown in **Fig. S19**. Furthermore, the AI D^2^NN can be expanded to visible or near-infrared spectral range for broader applications, with future developments expected to optimize factors such as energy efficiency, optical loss, fabrication precision, and process feasibility, major challenges are listed in **Table S6**.

**
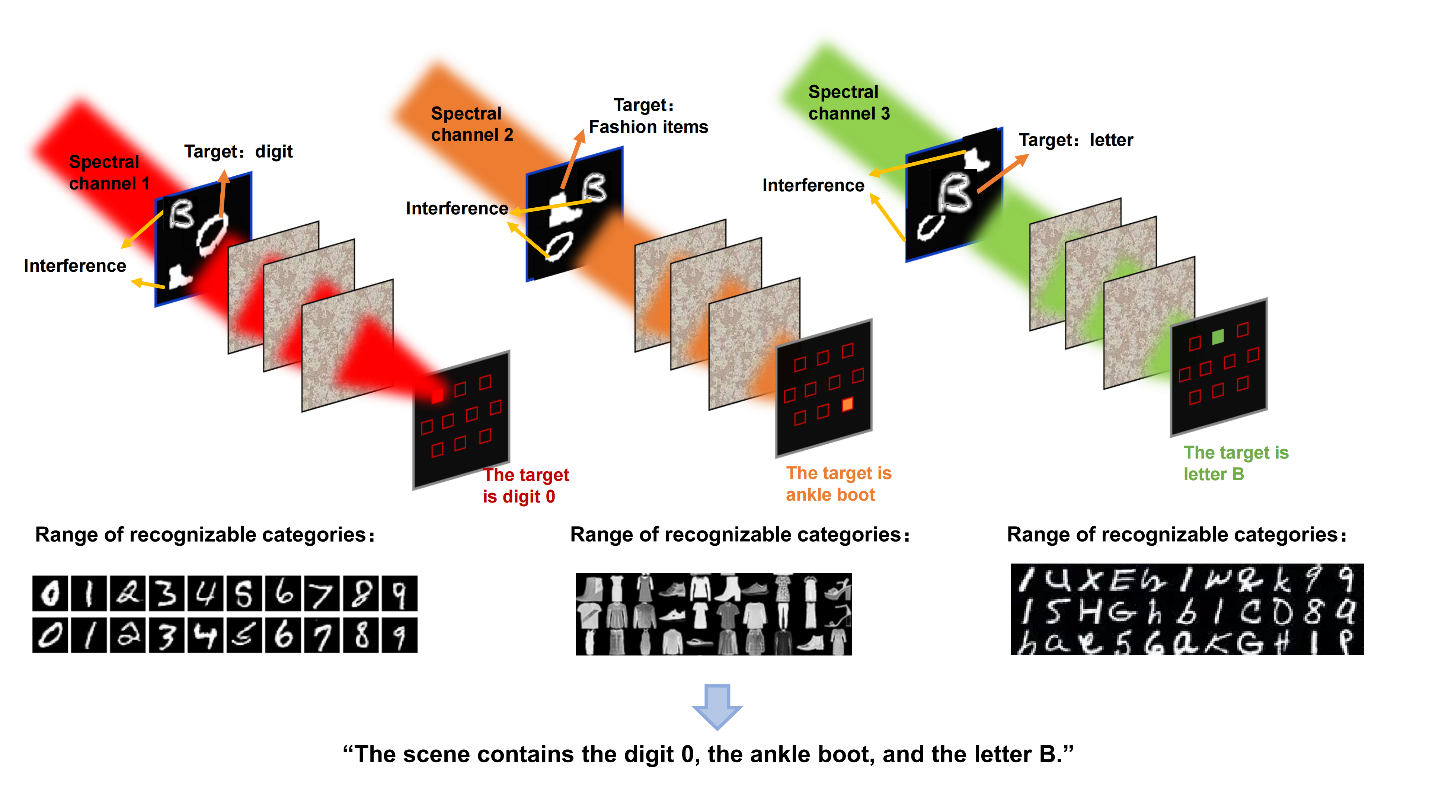
**

**Fig. S19 AI D^2^NN for** **multi-object classification in complex scenes**

**Table S6. Challenges of Metasurfaces across Different Spectral Bands**

| **Wavelength**  **Band** | **Materials** | **Challenges** |
| --- | --- | --- |
| Visible  (400–700 nm) | TiO₂, Si₃N₄ | 1. Limited tunability of material refractive index.  2. High fabrication precision (< 10 nm) |
| Near-Infrared  (700–1100 nm) | Si | 1. Absorption loss  2. Fabrication precision (~10 nm)  3. High efficiency  4. broadband response |
| Mid-Infrared  (2–20 µm) | Ge, GaAs,  VO₂, GST | 1. Material thermal stability  2. Absorption loss  3. Broadband phase modulation  4. Device lifetime |
| Microwave  (1-300 GHz) | Cu, Al,  Graphene,  Liquid crystal | 1. Device footprint  2. System integrability,  3. Dynamic tuning speed,  4. Electromagnetic loss of materials |

**Movie S1.**

The network’s classification capability under dynamic scenes

**Movie S2.**

The network’s classification capability under complex scenes

**References**

1. Lin X.*, et al.* All-optical machine learning using diffractive deep neural networks. **361**, 1004-1008. <https://doi.org/doi:10.1126/science.aat8084> (2018)

2. Kulce O., Mengu D., Rivenson Y., Ozcan A. All-optical synthesis of an arbitrary linear transformation using diffractive surfaces. *Light Sci Appl* **10**, 196. <https://doi.org/10.1038/s41377-021-00623-5> (2021)

3. Shen C.-Y.*, et al.* Broadband unidirectional visible imaging using wafer-scale nano-fabrication of multi-layer diffractive optical processors. *Light Sci Appl* **14**, 267. <https://doi.org/10.1038/s41377-025-01971-2> (2025)

4. He J. W., Wang X. K., Xie Z. W., Xue Y. Z., Wang S., Zhang Y. Reconfigurable terahertz grating with enhanced transmission of TE polarized light. *APL Photonics* **2**. <https://doi.org/10.1063/1.4986505> (2017)

5. Guo Z.*, et al.* Polarization-selective unidirectional and bidirectional diffractive neural networks for information security and sharing. *Nat Commun* **16**, 4492. <https://doi.org/10.1038/s41467-025-59763-6> (2025)

6. Duan Z., Chen H., Lin X. Optical multi-task learning using multi-wavelength diffractive deep neural networks. *Nanophotonics (Berlin, Germany)* **12**, 893-903. <https://doi.org/10.1515/nanoph-2022-0615> (2023)

7. Kulce O., Mengu D., Rivenson Y., Ozcan A. All-optical information-processing capacity of diffractive surfaces. *Light Sci Appl* **10**, 25. <https://doi.org/10.1038/s41377-020-00439-9> (2021)

8. Bai B.*, et al.* To image, or not to image: class-specific diffractive cameras with all-optical erasure of undesired objects. *eLight* **2**, 14. <https://doi.org/10.1186/s43593-022-00021-3> (2022)
